# Modeling cell populations metabolism and competition under maximum power constraints

**DOI:** 10.1371/journal.pcbi.1011607

**Published:** 2023-11-08

**Authors:** Luigi Conte, Francesco Gonella, Andrea Giansanti, Axel Kleidon, Alessandra Romano

**Affiliations:** 1 Department of Environmental Sciences, Informatics and Statistics, Ca’ Foscari University of Venice, Venezia Mestre, Italy; 2 Department of Physics, Sapienza University of Rome, Roma, Italy; 3 Centre for the Study of the Systemic Dynamics of Complex Diseases, Venezia Mestre, Italy; 4 Department of Molecular Sciences and Nanosystems, Ca’ Foscari University of Venice, Venezia Mestre, Italy; 5 THE NEW INSTITUTE Centre for Environmental Humanities (NICHE), Venezia, Italy; 6 Istituto Nazionale di Fisica Nucleare, Roma, Italy; 7 Biospheric Theory and Modeling Group, Max Planck Institute for Biogeochemistry, Jena, Germany; 8 Department of General Surgery and Medical-Surgical Specialties, University of Catania, Catania, Italy; University of Southern California, UNITED STATES

## Abstract

Ecological interactions are fundamental at the cellular scale, addressing the possibility of a description of cellular systems that uses language and principles of ecology. In this work, we use a minimal ecological approach that encompasses growth, adaptation and survival of cell populations to model cell metabolisms and competition under energetic constraints. As a proof-of-concept, we apply this general formulation to study the dynamics of the onset of a specific blood cancer—called Multiple Myeloma. We show that a minimal model describing antagonist cell populations competing for limited resources, as regulated by microenvironmental factors and internal cellular structures, reproduces patterns of Multiple Myeloma evolution, due to the uncontrolled proliferation of cancerous plasma cells within the bone marrow. The model is characterized by a class of regime shifts to more dissipative states for selectively advantaged malignant plasma cells, reflecting a breakdown of self-regulation in the bone marrow. The transition times obtained from the simulations range from years to decades consistently with clinical observations of survival times of patients. This irreversible dynamical behavior represents a possible description of the incurable nature of myelomas based on the ecological interactions between plasma cells and the microenvironment, embedded in a larger complex system. The use of ATP equivalent energy units in defining stocks and flows is a key to constructing an ecological model which reproduces the onset of myelomas as transitions between states of a system which reflects the energetics of plasma cells. This work provides a basis to construct more complex models representing myelomas, which can be compared with model ecosystems.

## Author summary

Ecological interactions at the scale of cell populations are important to understand the emergent behavior of complex biological systems as diseases. Despite the extensive knowledge of the molecular and cellular details of biomedical systems, understanding the mechanisms behind the onset of Multiple Myeloma and predicting its dynamic evolution is a challenge. For diseases characterized by recurrent patterns and non-genetic plasticity, it is not possible to eradicate the mechanism that sustains cancer onset and evolution, namely they are defined as incurable. This mechanism is not typical of blood cancer itself, being a general emergent behavior of ecological systems as determined by the complex interactions between system’s elements and the environment. Unfolding the role of the competitive interaction between normal, cancerous cell populations and the micro-environment by quantifying the energetic constraints operating on them is of key relevance. Here, adopting an energetic approach, we show that it is possible to reproduce stylized patterns of Multiple Myeloma onset by means of a minimal stock-flow ecological model of competing normal and malignant plasma cell populations. Our results suggest the potential of this general approach to build more detailed models of Multiple Myeloma, as well as to study biological systems and diseases.

## 1. Introduction

Ecological interactions at the cellular scale have been recognized as key elements to understand and model cancer [1–5]. Despite the substantial amount of literature dealing with mathematical modeling of cancer and interacting cellular populations, a simple unifying framework encompassing simultaneously the ecological and the evolutionary dynamics of cancer is lacking [6–9], along with the fact that integrating dynamical models of biological systems with energetic principles remains a challenge [10,11].

In this work, we use a minimal energetic approach based on stock-flow diagrams and ordinary differential equations to model the metabolisms of antagonist cell populations competing for common limited resources as regulated by microenvironmental factors and internal cellular structures. Our study focuses on the application of this formulation to simulate the evolution of a specific blood cancer, called Multiple Myeloma (MM). We represent the system as a simple ecosystem by modeling the flows of available and dissipated energy in equivalent units of ATP. In principle, this allows to link the typical patterns observed in the evolution of myelomas with their irreversible behavior as formulated in terms of energy and dissipation.

Multiple Myeloma (MM) is a blood neoplasm growing due to uncontrolled proliferation in the bone marrow (BM) of neoplastic plasma cells (PCs). Once mutations accumulate, neoplastic PCs abrogate control by local tissue constraints, and their newly acquired individual fitness is determined by the Darwinian interactions of their phenotype with critical properties of their local environment that plays an active supportive role [12–14]. Normal and neoplastic PCs compete for space within the bone marrow [15], with neoplastic PCs able to overcome the micro-environment constraints to sustain their proliferation owing to their phenotypes [16]. This often results in an evolutionary advantage of malignant cells that relies on a metabolism that facilitates the uptake and incorporation of nutrients into the biomass [17–21]. In patients, myelomas emerge at different times relative to the malignancy of the disease that developed in a clinical classification of its different asymptomatic and symptomatic phases [22–26].

When stocks are measured in energy equivalent units, stock-flow models give an effective energetic representation of the dynamics of systems. The use of energy system diagrams allows to derive differential equations from symbolic language by modeling and estimating useful and dissipated energy flows. In the case of cell population systems, this can be done by measuring the cell stocks in ATP equivalent units (*ATPeq*). The unit of *ATPeq*, is a measure of the available energy stored in the system that can be used by cells to perform work. It is a measure of the chemical potential energy or free energy stored in stocks which grow according to energy balance equations. This approach is tightly connected with quantitative ecosystems ecology, and it has been extensively applied to understand the evolution of ecological systems in connection with classical ecological modeling [27,28].

In the stock-flow formulation, cell populations are represented as cells stocks which produce and consume ATP owing to a self-regulation mechanism which prevents unlimited growth, enables the maintenance of the biological structure and the existence of stable stationary states for the system. These features define the process of ATP production and biosynthesis as the minimal set of dissipative processes required to define systems diagrams and general mathematical models involving cells stocks. An additional stock of resources, internal to the system, is needed to represent self-regulation as feedback that limits growth by controlling the energy and resource inflow from the external microenvironment. The internal resource stock defines the carrying capacity for the system and its accessible steady states, by providing a mechanism for non-linearity and stability resulting in logistic growth of the cell stock, which also depends on intrinsic growth rate and turnover time. Logistic growth and its extension to two antagonist interacting populations are the mathematical models corresponding to the presented stock-flow formulation when using the classical notation of mathematical biology (derivation and mapping in Methods). The model parameters are estimated from the literature as the growth rates of normal and malignant plasma cells phenotypes and their proteome turnover times. The estimate of the carrying capacity by means of the average number of plasma cells in the bone marrow in units of *ATPeq* reflects a reasonable upper limit for the PCs stocks in energetic terms.

**Fig 1:**
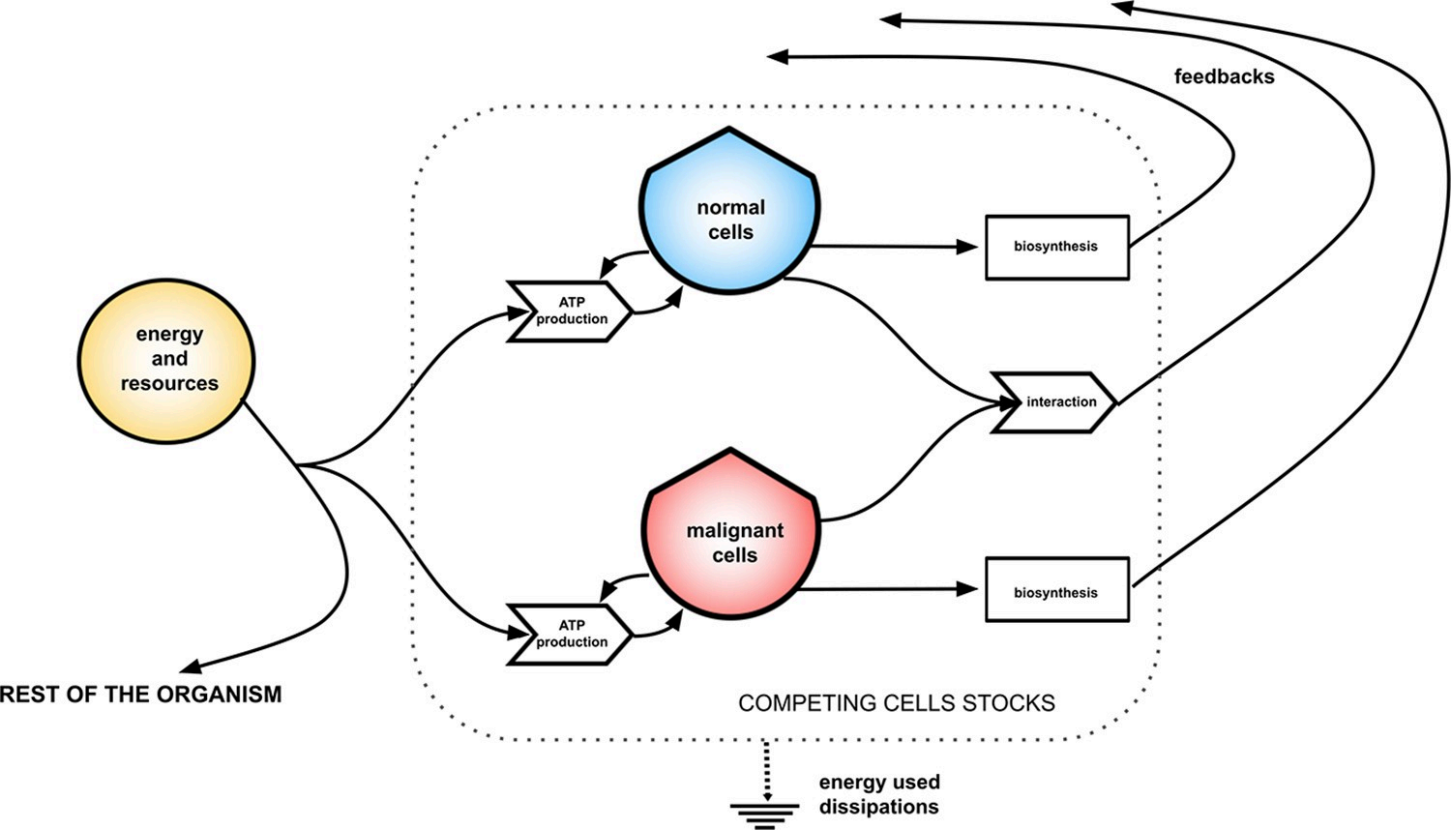
Energetic description of interacting cells stocks embedded in an organism. The diagram shows competing cells stocks as determined by the main process of ATP production, biosynthesis, and by the antagonistic interaction. It shows the associated flows of dissipated and useful energy, the latter being able to generate feedback for self-regulation on a higher hierarchical scale. The flow of energy and resources directed toward the rest of the organism is reduced by the inflows of the sub-system of competing cells stocks. The picture represents the interaction of plasma cells at myelomas onset which we define as being the *core* of the representation of Multiple Myeloma in the energy system language.

The model of two cells stocks competing for common limited resources imposes competitive exclusion [29]. This model system is thought to be a sub-system of a larger one representing the whole organism or the disease, as depicted in Fig 1. The malignant plasma cells stock outcompetes normal cells owing to their ability to incorporate energy and resources from the external microenvironment on a shorter time scale with respect to their normal counterpart, reflecting a more dissipative metabolism. By using single population estimates and by fine tuning the free parameter which varies the strength of the antagonistic interaction among the stocks, the simulations show a class of abrupt regime shifts leading to the extinction of the normal cells stock after a coexistence period with malignant cells which rapidly grow. This behavior, which is known from ecological models as regime shift [30,31], points out the existence of unstable stationary states of the system, which are observed for myelomas as an asymptomatic phase of the disease [22–26].The times for these transitions to occur are estimated in the range from few years to decades, consistently with clinical observations of the emergence of symptomatic disease.

Owing to the energetic approach and systems diagrams we define a minimal model able to reproduce a coarse-grained dynamics of myelomas evolution directly accounting for the metabolism of plasma cell populations, involving a small set of measurable parameters and, in principle, observable energy flows. The minimal model can be used to build more complex representations of myelomas. The transition to more dissipative states reduces the total available energy for the functioning of the whole system in which the competing cells stocks are embedded. It reflects the selective advantage of malignant cells as determined by the strength of the self-regulation feedback and by the strength of the antagonistic interaction which both set the timescale for the regime shift. The distance of the PCs steady states from a maximum power-dissipation limit, emerging as one of the possible stationary states compatible with self-regulation under mass balance constraints, is in principle an energetic measure of the evolutionary advantage of neoplastic PCs at the scale of the bone marrow. On the other hand, neoplastic cells appear to break down this regulation, acquiring the selective advantage needed to proliferate uncontrolled by means of more dissipative metabolisms.

Our approach is generalizable to the study of biological systems using symbolic energy language which allows the construction and the comparison of models at different spatial and temporal scales. By formulating the dynamics of systems in terms of energy and dissipation, and relating it to a physical limit, may help to identify general rules of organization in complex systems, no matter whether we look at the small scale of how cells interact within an organism, or at larger scales of plants and animals within an ecosystem, or the biosphere and the whole Earth system.

## 2. Results

### 2.1 The metabolism of normal and malignant plasma cells as stationary states

**Fig 2:**
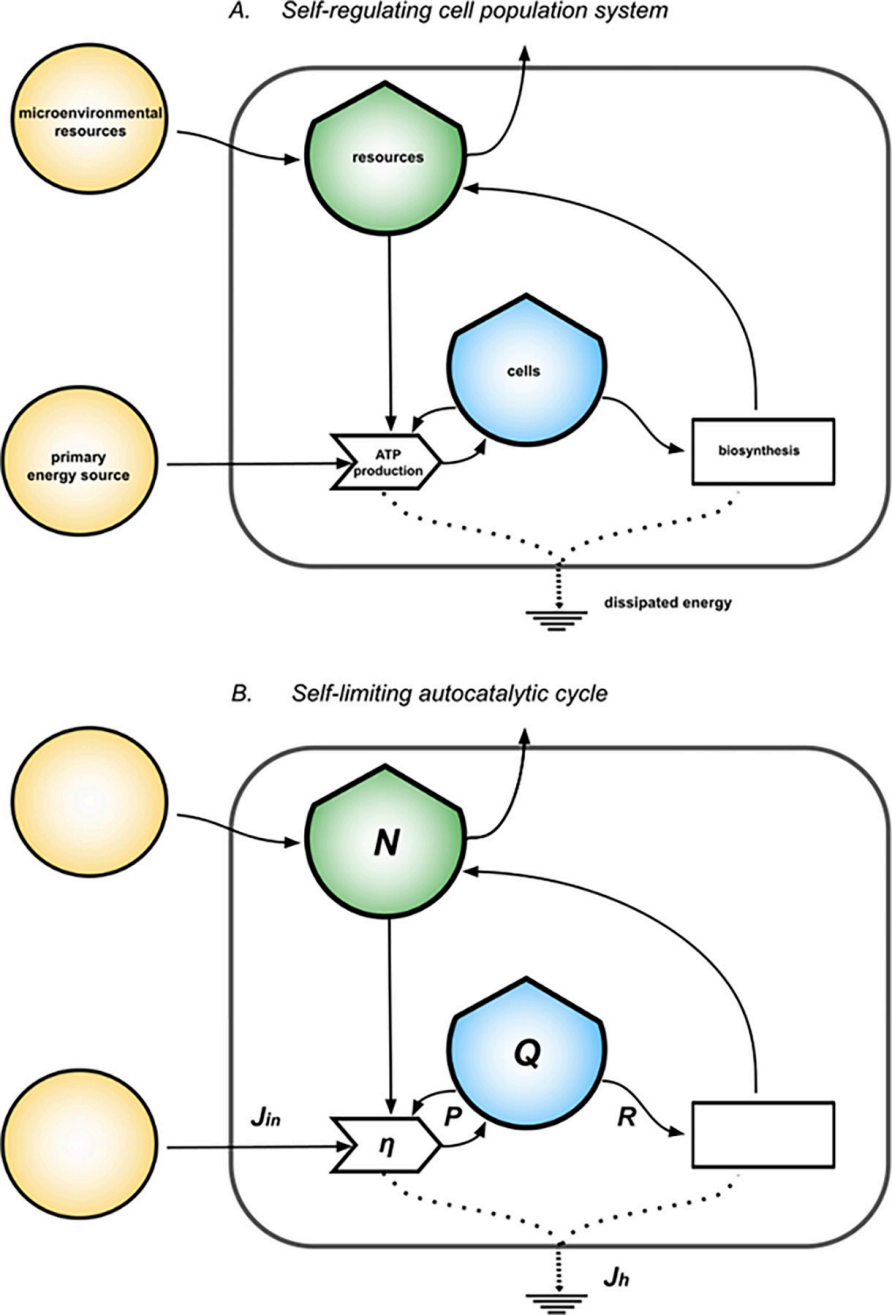
Self-regulating cell population system. The diagram in (A) represents a self-regulating cell population seen as a system that produces and consumes ATP from primary energy and microenvironmental resources, respectively through the processes of ATP production and biosynthesis. The cells stock exerts feedback on the ATP production process needed for proliferation. From the biosynthesis process develops feedback on growth represented by the population’s ability to: 1) regulate limiting factors coming from the interaction with the micro-environment, as cofactors and dissolved ions; 2) produce functional proteins, molecular machines and enzymes that enable reactions and control their rates. From (A) we define in (B) the modeled quantities: the primary energy inflow, *Jin*; the ATP production efficiencies, *η*; the cells stock *Q* that grows according to $\frac{dQ}{dt}=P-R$ with *P*(*Q*) = *ηJ*_*in*_ = *rQ*·(1−*Q*/*K*), net power inflow (with *r* intrinsic growth rate, *K* carrying capacity), and *R*(*Q*) = *Q*/*τ* energy outflow, being *τ* the proteome turnover time; the stock of resources, *N* = *K*−*Q*, for the mass balance to hold; the total heat flow *J*_*h*_ = *P*/*η* = *J*_*in*_ in steady state.

We model a system representing a self-regulating cell population with the self-limited autocatalytic cycle model explicitly accounting for mass and energy balance [28] as shown in Fig 2. The effective dynamics is logistic growth. In this formulation, the stock of resources *N* plays a central role of mass balance constraint providing the mechanism required for non-linearity and stability, as derived in Methods. If *Q(t)* is the stock of energy stored in a cell population measured in *ATPeq* at time *t*, then its change in time (*dQ/dt*) is given by the balance between the net ATP production inflow, *P(Q)*, and the *ATPeq* outflow driving biosynthesis, *R(Q)*, as:

$$\frac{dQ}{dt}=P(Q)-R(Q)=rQ\cdot (1-\frac{Q}{K})-\frac{Q}{\tau }$$ (Eq 1)

being *r* the intrinsic growth rate of *Q*, *K* the carrying capacity of the system and *τ* the stock turnover time from which the steady states depend as derived from Eq 1:

$$P_{ss}=\frac{Q_{ss}}{\tau }=\frac{K}{\tau }\cdot (1-\frac{1}{r\cdot \tau })$$ (Eq 2)

with *Pss* and *Qss* the steady state values for *P(Q)* and *Q*.

We model the stationary states of normal and neoplastic plasma cells populations with Eq 2 and classify them on the quadratic curves *P(Q)* and *Jh(Q)*, the latter modeling the total dissipative heat produced by the system. The expression *Jh(Q)* is obtained by balancing the energy flows in the diagram in Fig 2 (details in Methods), assuming a general efficiency relation between the energy inflow *Jin* and the power *P* as *P* = *η*·*J*_*in*_, with *η* being the efficiency of ATP production, so that *J*_*h*_(*Q*) = *P*(*Q*)/*η*. The maximum of *P* represents the maximum rate at which energy and resources can be converted and stored in the cell stocks given *r*, *K* and *τ*, resulting in the maximum rate of energy dissipated. We evaluate the stability of the system by means of the analytical stability potential *V(Q)* derived using the physical concept of scalar potential [32,33].

**Fig 3:**
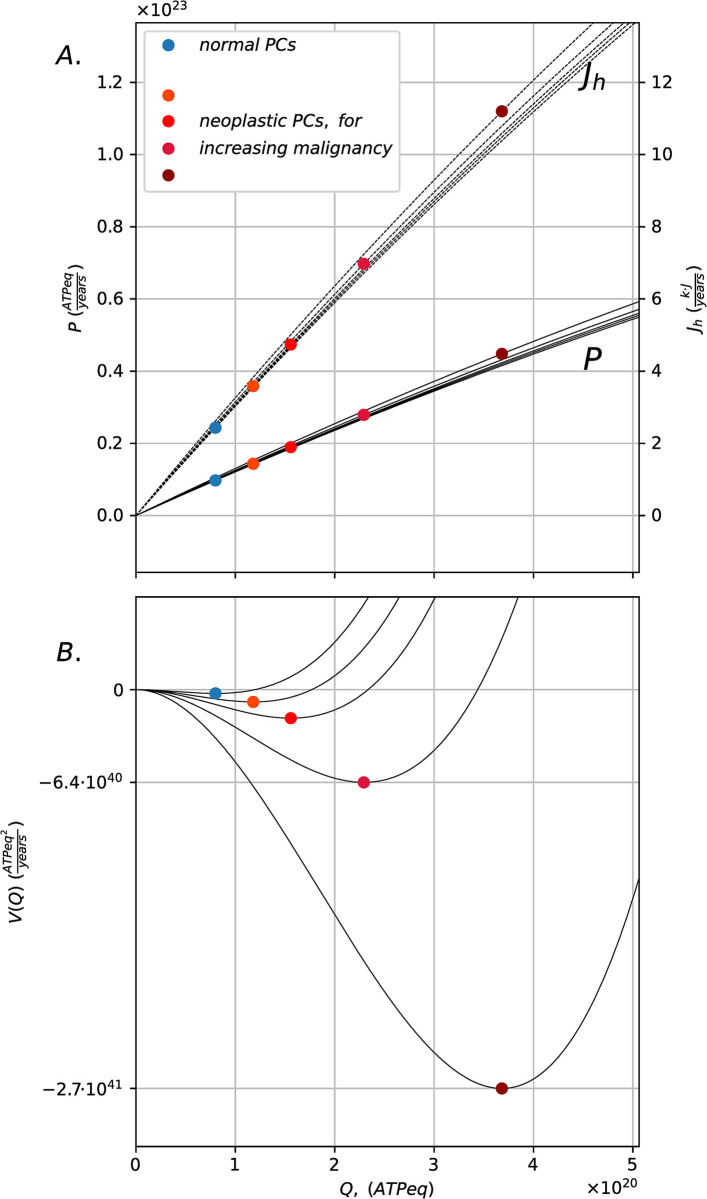
Stable stationary states for normal and neoplastic plasma cells. Panel (A) shows the actual steady states (colored dots) occupied by the PCs system in the state space defined by the bell-shaped curves *P*(*Q*) = *r*·*Q*·(1−*Q*/*K*) and *J*_*h*_(*Q*) = *P*(*Q*)/*η*, depending on the intrinsic growth rates *r* of normal PCs (*r* = 1.39·10^−2^*h*^−1^, in blue) and malignant PCs (*r* = 1.43·10^−2^*h*^−1^−1.53·10^−2^*h*^−1^, red hues), with *K = 4*.*05·10*^*21*^ *ATPeq*, *τ = 72 h* (parameter estimation in Methods). Panel (B) shows the associated stability curve *V(Q)* defined by *dV*(*Q*)/*dQ* = −*dQ*/*dt* (Eq 8, derived in Methods).

The stationary states that model the metabolisms of normal and neoplastic plasma cell populations according to Eq 2 are shown in Fig 3. The parameters in Eq 2 are estimated through reported indirect measures of intrinsic growth rates and proteome turnover times of plasma cells. The carrying capacity is estimated from the average number of cells which can be plasma cells in the bone marrow, measured in *ATPeq*. The efficiency of ATP production is estimated accounting for the pathway of oxidative phosphorylation. The blue dot identifies the stationary state for normal PCs, while the dots of red hues identify the stationary states for neoplastic PCs for phenotypes of increasing malignancy, identified in the model by a greater growth rate. Increases in growth rates lead to higher stock values that have access to higher power inflows corresponding to higher dissipative heat flows. These states are more stable the greater the stock value, according to the logistic growth model. Among all phenotypes of PCs, normal PCs occupy the steady state corresponding to the smallest stock value, the lowest ATP production flow, the lowest dissipative heat flow, and the least stability, as compared to neoplastic PCs in shades of red. Neoplastic plasma-cells set on pathways of increasing power generation, increasing dissipations, and increasing stability for increasing malignancy. Malignant PCs populations have a higher ability to convert the primary energy inflow into useful power to grow population structure. This corresponds, in the model, to more dissipative and stable metabolisms. The absolute values of power generation (and heat flows) are comparable among all the phenotypes and range within the same order of magnitude.

The increasing ability of neoplastic PCs in generating useful power by increasing malignancy—parametrized by the growth rates—reflects the general biomedical observations of myeloma onset at the level of the bone marrow. In our model, given the constraints of resources availability and the estimated growth regimes, increasing power generation corresponds–at the same time–to:

- increasing ability of neoplastic PCs populations to grow, incorporate nutrients and occupy space with respect to normal PCs, characterized by increases in *Q* and decreases in *N* (being *N* = *K*−*Q*);
- increasing ability to absorb external primary energy inflow *Jin*, being *P* = *η*·*J*_*in*_ with fixed *η*, corresponding to increasing dissipations in the steady state, as *J*_*h*_ = *J*_*in*_.

Increases in the primary energy inflow, in the incorporation of nutrients and in the occupation of space in the bone marrow reflect the ability of neoplastic PCs to change the micro-environmental constraints operating on the system in advantage of their growth. Moreover, malignant cell states appear to be more dissipative and stable providing, in principle, an energetic measure for their selective advantage.

### 2.2. Scenarios of uncontrolled growth of malignant cells

**Fig 4:**
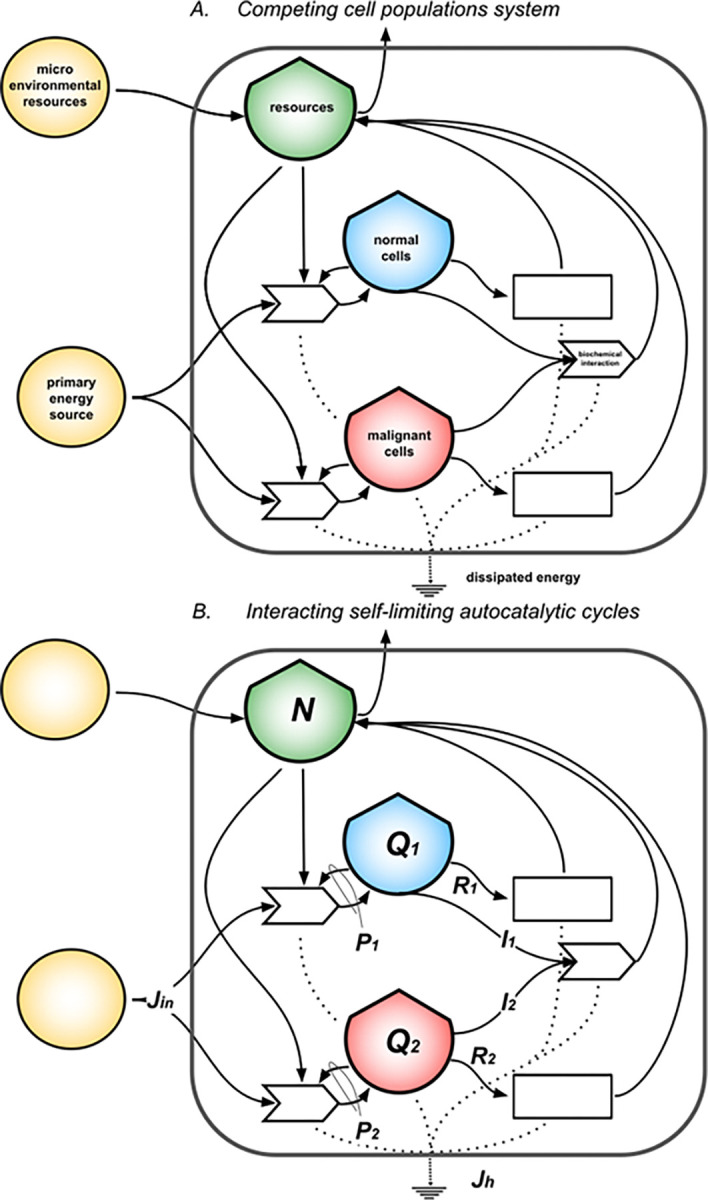
Competing cell populations system. The diagram in (A) represents a system made of normal cells competing with its malignant (cancerous) counterpart for common limited resources. Each single population is seen as a system that produces and consumes ATP, following the model in Fig 2. In addition, populations compete for space in the tissue and interact by means of an effective biochemical interaction which favors the death of the competitor. Each process dissipates energy under the form of heat that sinks at the bottom of the diagrams. Panel (B) shows the mathematical model for the system. It highlights the physical observables: external primary energy inflow, *Jin*, the net power inflows, *P1* and *P2*, for the respective cells’ stocks, *Q1* (normal PCs) and *Q2* (neoplastic PCs), and the heat flow, *Jh*, the stock *N* of resources (shared by *Q1* and *Q2*); the biochemical antagonistic interaction is parametrized by *I1* and *I2*.

We model two cell populations competing for common resources as a simple ecological system composed of two interacting metabolisms [28] as shown in Fig 4. The extension of Eq 1 for two cells stocks is given by the system of coupled differential Eqs 3 and 4. If *Q1(t)* and *Q2(t)* represent stocks of energy stored in the populations of cells measured in *ATPeq* at the time *t*, the changes in time of the stocks (*dQ1/dt* and *dQ2/dt*) are given by the balance equations:

$$\frac{dQ_{1}}{dt}=P_{1}(Q_{1},Q_{2})-R_{1}(Q_{1})-I_{1}(Q_{1},Q_{2})=r_{1}Q_{1}(1-\frac{Q_{1}}{K}-\frac{Q_{2}}{K})-\frac{Q_{1}}{\tau_{1}}-\alpha_{1}Q_{1}\cdot Q_{2}$$ (Eq 3)

$$\frac{dQ_{2}}{dt}=P_{2}(Q_{1},Q_{2})-R_{2}(Q_{2})-I_{2}(Q_{1},Q_{2})=r_{2}Q_{2}(1-\frac{Q_{1}}{K}-\frac{Q_{2}}{K})-\frac{Q_{2}}{\tau_{2}}-\alpha_{2}Q_{1}\cdot Q_{2}$$ (Eq 4)

being *P1*, *P2* the net ATP production inflows, *R1*, *R2* the *ATPeq* outflows driving biosynthesis, *I1*, *I2* the biochemical antagonistic interaction respectively for the stocks *Q1* and *Q2*, and being *r1*, *r2* the intrinsic growth rates, *τ1*, *τ2* the turnover times, *α1*, *α2* the strength of the biochemical interaction, respectively for the stocks *Q1* and *Q2*, and being *K* the carrying capacity of the system. The model has 2 non-trivial stationary states *(a)* and *(b*) for the stocks given by:

$${Q_{1,ss}}^{\left(a\right)}=K\cdot (1-\frac{1}{r_{1}\cdot \tau_{1}}),{Q_{2,ss}}^{\left(a\right)}=0$$ (Eq 5)

$${Q_{2,ss}}^{\left(b\right)}=K\cdot (1-\frac{1}{r_{2}\cdot \tau_{2}}),{Q_{1,ss}}^{\left(b\right)}=0$$ (Eq 6)

The state *(a)* is unstable with respect to small perturbation with the parameter setting representing normal and neoplastic plasma cells in competition, while the state *(b)* is stable. This provides the conditions for the transition of the system from the initial state *Q*_1,0_ = *K*·(1−1/*r*_1_·*τ*_1_) and *Q*_2,0_ = 5·10^10^ *ATPeq* to the stable stationary state *Q*_1,*ss*_ = 0 and *Q*_2,*ss*_ = *K*·(1−1/*r*_2_·*τ*_2_). The unstable initial condition represents the time the first mutation of one single plasma cell occurs with normal cells in steady state in the bone marrow. The final stable state represents the extinction of normal cells due to the uncontrolled proliferation of malignant cells.

The total heat flow *Jh(Q1*,*Q2)* is derived by balancing the energy flows in the diagram in Fig 4 as shown in Methods. In steady state, *J*_*h*_(*Q*_1_, *Q*_2_) = *P*_1_/*η*_1_+*P*_2_/*η*_2_, being *η1* and *η2* the efficiencies of ATP production for *Q1* and *Q2*.

**Fig 5:**
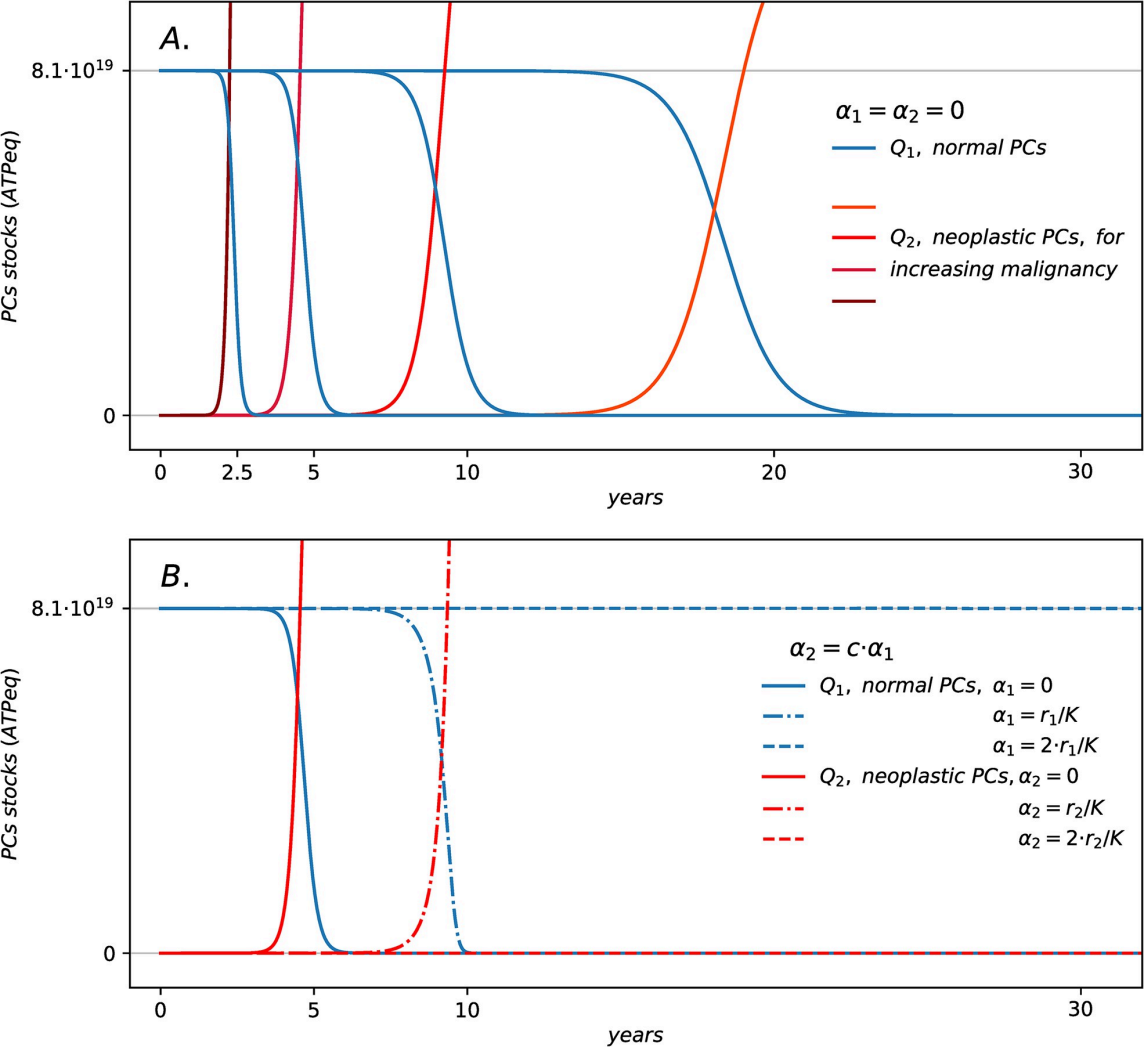
Coexistence and regime shifts for interacting normal and neoplastic plasma-cells. Panel (A) shows the time evolution of stock *Q1* (in blue) and *Q2* (in red hues) according to Eqs 3 and 4 on yearly time scale for configurations defined by the different values of *r*_2_ = 1.43·10^−2^−1.53·10^−2^*h*^−1^ for neoplastic PCs as in Fig 4, and with *K = 4*.*05·10*^*21*^ *ATPeq*, *r*_1_ = 1.39·10^−2^*h*^−1^, *τ1 = τ2 = 72 h*, *α1* = *α2* = 0, *Q1(0) = Qss = 0*.*02·K*, *Q2(0) = 5·10*^*10*^ *ATPeq*. Panel (B) shows the time evolution of stock *Q1* (blue) and *Q2* (red) for configurations defined by the different values of *α1 = c·r1/K* and *α2 = c·r2/K* with *c = 0*, *1*, *2* (solid, dashed, dot dashed lines) and by *r*_2_ = 1.47·10^−2^*h*^−1^, *r*_1_ = 1.39·10^−2^*h*^−1^, *K = 4*.*05·10*^*21*^ *ATPeq*, *τ1 = τ2 = 72 h*, *Q1(0) = Qss = 0*.*02·K*, *Q2(0) = 5·10*^*10*^ *ATPeq*.

The numerical integration of Eqs 3 and 4 provide minimal dynamics of Multiple Myeloma onset based on the competition between normal (*Q1*, in blue) and neoplastic plasma cells (*Q2*, in the red hues) in the bone marrow. By setting up the parameters defining the steady states for normal and malignant plasma cells and by fine tuning the parameters *α1* and *α2*, we derive stylized scenarios of myelomas evolution. The transition is due to a mutation of one normal cell perturbing the stationary state of the system modeled by the initial conditions, as shown in Fig 5.

Fig 5 shows the irreversible regime shifts for the stocks for different values of growth rate of the malignant cells. After a coexistence period between an unperturbed normal cells stock with a slowly growing malignant one, the originally adapted plasma-cells population rapidly extinguishes, while the malignant cells grow within the bone marrow, exceeding the original steady state. It can be shown from the simulations that the patterns for the ATP production flows, and the total heat flow resemble the regime shift dynamics, following an abrupt growth due to neoplastic PCs growth (see Methods). The coexistence times, and thus the critical time for the regime shift, depends in turn on the growth rates of the malignant population. The faster neoplastic PCs grow, the shorter the coexistence time with normal PCs, and the sooner the regime shift occurs with respect to the time of the first mutation.

The trajectories in Fig 5B show the dynamics of the system for configurations defined by fixed growth rates (with neoplastic PCs advantaged over normal ones) and different values of the strengths of *I1* and *I2*. The initial conditions are the same as for Fig 5A, and so for the carrying capacity and the turnover times. Fig 5B shows that, depending on the strength of *I1* and *I2*, the system undergoes or not a regime shift within the time window considered. The regime shift patterns in Fig 5B are analogous to the one in Fig 5A in terms of the dynamics of stocks and flows. In these configurations, the stronger the interaction the longer is the critical time for the regime shift. Fig 5B also shows that increasing the interaction strength can lead to survival of the originally adapted and more abundant population, although the malignant population is advantaged by its growth regime. In this case, normal PCs survive the perturbation of neoplastic ones by maintaining their steady state. The interaction terms *I1* and *I2* act as delays of the overall dynamics because these are terms that deplete both stocks (appear in Eqs 3 and 4 with the minus sign), reducing the values for both *Q1* and *Q2* and thus indirectly reducing the growth rates, slowing down the dynamics. This explains why the regime shift time is longer the stronger the antagonistic interaction. In this sense, *I1* and *I2* measure an additional free energy cost for the competing stocks to sustain antagonistic interaction by means, for example, of apoptosis control. Overall, the timescale of the regime shift is set by the depletion of the stock *Q1*, thus it depends on all flows and on the initial conditions. It is a systemic property of the configuration, which indirectly depends on the parameters that define the flows as well as on the initial values of the stocks.

## 3. Discussion

We provide a self-consistent approach to model cellular systems that is based on energy system diagrams and the associated ordinary differential equations. We focus on the application of this approach to simulate the onset of a blood cancer called Multiple Myeloma, as determined by the uncontrolled proliferation of cancerous plasma cells in the bone marrow. In this work, we reproduce the stylized patterns observed in Multiple Myeloma evolution with a minimal competition model accounting for the basic metabolism and phenotypic traits of plasma cells. In the model, neoplastic PCs use in a different way elements, processes and flows that already exist in their normal counterpart (corresponding to a different parameter setting). Genetic mutations create the conditions for the system to access states of uncontrolled proliferation of malignant cells by perturbing the stationary states of normal cells (translated into the initial conditions of the system dynamics). The dynamics of the model is characterized by irreversible shifts of regimes that lead to the extinction of normal plasma cells. The scenarios are consistent with biomedical knowledge and observations of the different stages and emergence of the MM disease [22–26]. The model parameters and flows are, in principle, measurable by clinicians.

The presented approach can capture the complexity of a system in terms of the feedback network’s dynamics, allowing it to represent the system as the network of energy flows that ultimately determines its resilience, stability and reaction to external perturbations. We apply this to study the evolution of a biomedical system exhibiting highly complex behaviors, such is the case of Multiple Myeloma. In this sense, the capacity for generating new insights about cancer evolution derives from the novelty of the language it uses, that describes a complex disease starting from the description of functional units connected by real flows, all measured using the same energetic unit. Stock-flow systemic representation has the capability of considering and estimating thermodynamic quantities, including heat dissipation. Most of all, since the network of feedback is what ultimately determines the dynamics of the system, a final comprehensive diagram can effectively capture it, thus offering predictive responses to the "what if" questions.

### 3.1 Energetic constraints and the metabolism of plasma cells

The interaction between the cell stocks and the micro-environment sets the constraints on the dynamics of both single and competing PCs populations. In our models, the microenvironment plays a central role in terms of systems dynamics. It represents the source of non-linearity that forces stocks to self-organize to survive on limited resources, by developing a self-regulating feedback that controls growth through the internal stock *N*. This mechanism generates a trade-off between the limited available resources and the population biomass from which the existence of the maximum power state derives. This state represents the maximal rate at which energy and resources can be converted into biomass. Using *N* as a single stock of limiting factors is an approximation based on the system description in energy units and the scale, we choose to model the system, which is the scale of the bone marrow tissue. This is reasonable so far, we want to describe the very basic control mechanism that decouples the cellular system from the external environment by providing the adaptive mechanism needed to reach a steady state through self-regulation. This is indeed a first-step description which depends on the level of abstraction of the model, but already captures the dynamics of what can be observed, namely competitive advantage. The aim of the representation is to provide a constraint which is general enough to be compatible with growth and can address a representation of limiting factors needed to benchmark more complex ones. Moreover, the limited availability of resources imposes a constraint on the abundance of interacting populations that controls the conditions for coexistence and survival of the two. In this formulation, with similar growth rates, only the population that is faster in resource acquisition (power generation) survives in the long run, however, this inevitably requires a more dissipative metabolism that reduces the total available energy for the larger system in which the competing cells are embedded.

The stationary states of normal and neoplastic plasma cells are represented as steady states of the self-limiting autocatalytic cycle model, which provides a minimal model of growth that accounts for the basic mechanism for self-regulation of cells and their energetics, as measured by ATP equivalent units (*ATPeq*). The model parametrizes logistic growth of a cells stock which only depends on two measurable typical timescales, namely, the relative growth rate of the cell population and its proteome turnover time. By estimating the parameters for the different phenotypes of normal and cancerous plasma cells, we show that, for increasing malignancy, neoplastic plasma cells set on more dissipative and stable states, reflecting a breakdown of regulation at the scale of the bone marrow which, according to the competition model, drives the onset of Multiple Myeloma. In our formulation, owing to a change in the growth rate, malignant cells are able to incorporate more nutrients into biomass by changing the boundary conditions for the system dynamics, namely the energy and resources inflow, in line with the biomedical phenomenology [17–20]. In the model, higher energy and resource inflows correspond to greater dissipations, reflecting open boundary conditions and a more dissipative metabolism for neoplastic PCs, an hypothesis which may be tested through the observation of heat shock proteins in neoplastic plasma cells [34] connected with measures of temperatures and heat flows [35]. Notably, in Fig 3, the growth regimes of single normal and malignant populations are differently located along the power-dissipation curves derived from the energy diagrams, by providing, in principle, an energetic measure of the evolutionary advantage of cancerous plasma cells, based on a small number of observable parameters and real flows. Despite phenotypic diversity, normal and malignant PCs appear to operate in a narrow portion of the state space, suggesting that the conditions for the survival of PCs are significantly constrained by the microenvironment on which they depend. This ultimately results from the fact that the typical time for which PCs protein synthesis reactions take place does not change upon accumulation of genomic alterations for malignant plasma cells [36]. What changes is the strength of the related resource flows, due to the interaction of cells with the microenvironment. This result is in line with the biomedical literature highlighting the fundamental active role of the bone marrow in PCs evolution [12–14].

### 3.2 Competition and evolution in Multiple Myeloma

We represent the onset of Multiple Myeloma as a class of dynamical transitions from the stable stationary state of normal plasma cells to more dissipative states of proliferating cancerous plasma cells, eventually causing the extinction of the normal ones. These patterns of uncontrolled proliferation of neoplastic plasma cells are modeled by extending the self-limiting autocatalytic cycle to interacting normal and neoplastic plasma cells competing in the bone marrow. The dynamics of this system is governed by the competitive Lotka-Volterra model. The transitions reflect a breakdown of self-regulation in the bone marrow as measured by the increased dissipations, namely, the total energy used in the system estimated by balancing the energy flows in the diagram of Fig 4. The model is characterized by the presence of unstable stationary states for the system, which are observed for myelomas as an asymptomatic phase of the disease [22,26]. The times for these transitions to occur are estimated in the range from few years to decades, consistently with clinical observations of the emergence of symptomatic disease. The mathematical model supporting our description is minimal, yet the use of ATP equivalent energy units in defining stocks and flows is a key to constructing an ecological model which reproduces reasonable timescales for the onset of Multiple Myeloma. In this sense, the energetic accounting of plasma cells may provide a building-block description to construct more complex models representing the whole system. The interaction terms act as delays of the overall dynamics as these deplete both energy stocks by indirectly reducing their growth rates, explaining why the regime shift time is longer the stronger the antagonistic interaction. In this sense, the flows measure an additional free energy cost for the competing stocks to sustain antagonistic interaction, for example, by means of apoptosis control between plasma cells which interact through the synthesis of antibodies and cytokines.

The differences in phenotype of plasma cells may be used as a diagnostic to build scenarios for the evolution of Multiple Myeloma, if supported by clinical observations. In the case of Multiple Myeloma, slow growing neoplastic PCs are known to be at the origin of asymptomatic manifestations of the disease, linked to their long coexistence periods with normal PCs. On the other hand, faster growing ones lead to symptomatic states with an abrupt and irreversible emergence of the disease [23–26]. Moreover, the proposed taxonomy can be in principle validated by cytometry experiments routinely performed by clinicians. Model validation schemes based on laboratory experiments have to be designed to fully address the potential of the formulation. Flow cytometry, calorimetric techniques and proteomics may be valuable approaches to make an advance in hypothesis testing [35]. This may give the possibility to develop ecology inspired early warning systems–based on dynamical systems simulations and energetic measures–able to support prognosis formulation, risk analysis and clinical decision making, as also suggested by (5) and (1).

It is worth underlining that the energy systems language plays in our modeling a central epistemic role. Indeed, stock-flow diagrams:

- Allow to determine the set of coupled differential equations necessary to create a computational simulator, starting from the symbolic language of energetics. We think that this perspective has great potential in bridging the community of modelers, clinicians and biologists, by providing an established and (in principle) common language able to make advances in research starting from knowledge of biological and biomedical systems without the specific knowledge of mathematics.
- Allow to consider and estimate thermodynamic quantities, in particular, dissipations (heat flows), which play a major role in ecological systems and that are typically not considered in more classical mathematical biology and mathematical oncology approaches.
- Capture the dynamics of the system, since diagrams are not some kind of static “photograph” of it, but rather a representation of the network of feedback operating at different scales which ultimately determine the system behavior.

In perspective, our approach may:

- In the simulation procedure, allow to localize the action of drugs and therapeutic strategies, even at different times of administration.
- Can be studied as the basis for simulating the response to the “what if” questions, making them a potentially important tool for the study of the role of both environment and therapies.
- Address positive and negative systemic leverage points, thanks to the sensitivity analysis carried out by the simulators.

It is finally worth underlining those minimal models–such as for example self-limiting cycles, balancing feedback, stocks local competitions, etc. are commonly used in Systems Thinking diagramming as dynamics functional units (building blocks) for setting up a more complex coherent description of the overall dynamics [28,37]. Our resulting model, despite its apparent simplicity, is not a standard one. It is not aimed at studying the evolution of specific cell populations, but rather the overall evolution of a system that we identify with the disease. Differently from existing approaches, in our formulation the mathematics of cancer evolution emerges by our description of how the complex system we identify as “the core” of the disease manages the real flows exchanging energy between the stocks, through processes that are in turn ruled by virtually all the other stocks entering in the disease definition.

### 3.3 Future development

Indeed, the development of more comprehensive models requires a systematic reference to actual observations. Accounting for more than two interacting populations, to represent diversity in PCs phenotypes as well as the interactions with other cells, and having a more refined representation of multiple limiting factors, which may influence growth on different time-scales, will be a necessary and natural extension of the present formulation, in order to characterize the evolution of Multiple Myeloma, as an example of complex disease with multifactorial etiopathology. To enhance the representation of the dynamics of cell population systems, a stochastic formulation of the derived non-linear dynamical equations can be included, to account for the uncertainty in the parametrization of the flows and in the evolutionary processes that shape cell phenotypes and so the model parameters.

The original view of tumor metabolism proposed by Warburg in 1927—when he suggested that “The resistance of single tumor cells is not to be compared with that of single normal cells, but rather the tumor as a whole with the organism as a whole”—helps as well to frame this study in a systems perspective. In the last years, the application of systemic approaches in biology and medicine has drawn considerable attention. Nevertheless, they are mostly based on computational tools using big data sets to perform Network Analysis procedures. Concepts like *system biology* [38–41], *bioinformatics* [42], *network medicine* [43,44], *network pharmacology* [45], *deep learning* [46], are used for example in several fields of biomedical research, often interpreting incurable diseases as some network perturbation [47] to be addressed by computational means [48,49]. These approaches are somewhat limited, being for instance unable to address the origin of “The enormous non-genetic plasticity of tumor cells” [50]. The need for a novel perspective has been claimed in recent years by several scholars [8,9], the latter claiming that “We urge a rethinking of systems biology as it develops toward systems medicine.… We need to balance the pathway-centric approach, focusing on cellular mechanisms, by ‘zooming out’ to actively seek law-like principles”. Indeed, the complementarity between Systems Thinking and Network Analysis has a strong potential.

The approach proposed here is at the same time abstract in nature and open to experimental observations and activity. It is worth mentioning that the Systems Thinking approach has been recently used by some of the Authors to describe the dynamical configuration of the virus-host interaction in the case of Covid-19 contagion [51]. In this framework, SARS is not classifiable as a complex disease, but the Systems Thinking description allows quantitative studies–by means of a computational simulator–on how the effectiveness of different therapeutic strategies may differ significantly depending on the time schedule of administration. We emphasize what we think is the real novelty of the presented approach: the system to be studied is not the cancer cell, the genome, the tissues, the organs, the patient or the set of patients, but the disease itself. From the study, a possible representation of complex disease is provided as the set of elements inside the boundary of a diagram able to self-organize in a subset of specific configurations that represent what we call Multiple Myeloma (in case of plasma cell populations). This is because *the disease is regarded as a systemic property* or *dynamical pattern*. So, we can explore–*inter alia*–why some people do not develop organ damage, staying without symptoms for all their lives (the patterns characterized by coexistence of normal and few malignant plasma cells), since the configuration will be that of the disease, but in a state for which the pattern of feedback allows to prevent clinical symptoms from occurring (before the system undergoes a regime shift). It is therefore a new way to describe the disease, since it integrates the biochemical degrees of freedom with which it is born and develops, and the details of what locally acts in the system. On the other hand, this approach allows to understand how and why the disease may proceed, right because we study the dynamics of the state configurations, expressed by cells stocks that are in principle measurable and inevitably subjected to thermodynamic constraints.

The picture of myelomas emerging from this work is that the distance of the stationary states of plasma cells from a maximum power-dissipation limit, as one of the possible steady states compatible with self-regulation under mass balance constraints, is in principle an energetic measure of the evolutionary advantage of neoplastic plasma cells at the scale of the bone marrow. At the scale of the whole organism, in the absence of cancerous cells, normal PCs occupy steady states of least dissipation among the PCs phenotypes, due to a a self-regulating control of metabolism which suggests a maximization of power on a larger temporal and spatial scale, possibly involving the immune system and the whole organism that we do not resolve with the actual model.

## 4. Methods

### 4.1 Derivation of model equations form systems diagrams

The state of the system at any time is defined by an *N*-ple of stocks that evolve according to a system of coupled differential equations given by

$$\frac{dQ_{k}}{dt}=f_{k}(Q_{1}\ldots Q_{N})=\sum_{i=1}^{n}J_{,k_{i}}-\sum_{j=1}^{m}J_{out,k_{j}},\;\mathrm{with}\;k=1,\ldots N$$ (Eq 7)

with *fk(Q1*,…*QN)* being in principle a nonlinear model function of *Q1…*.*QN*, that depends on the inflows *Jin*,*k*_*i*_ and the outflows *Jout*,*k*_*j*_ for the stock *Qk* (with *i = 1*,…*n* and *j = 1*,…*m* counting the number of flows).

**Fig 6:**
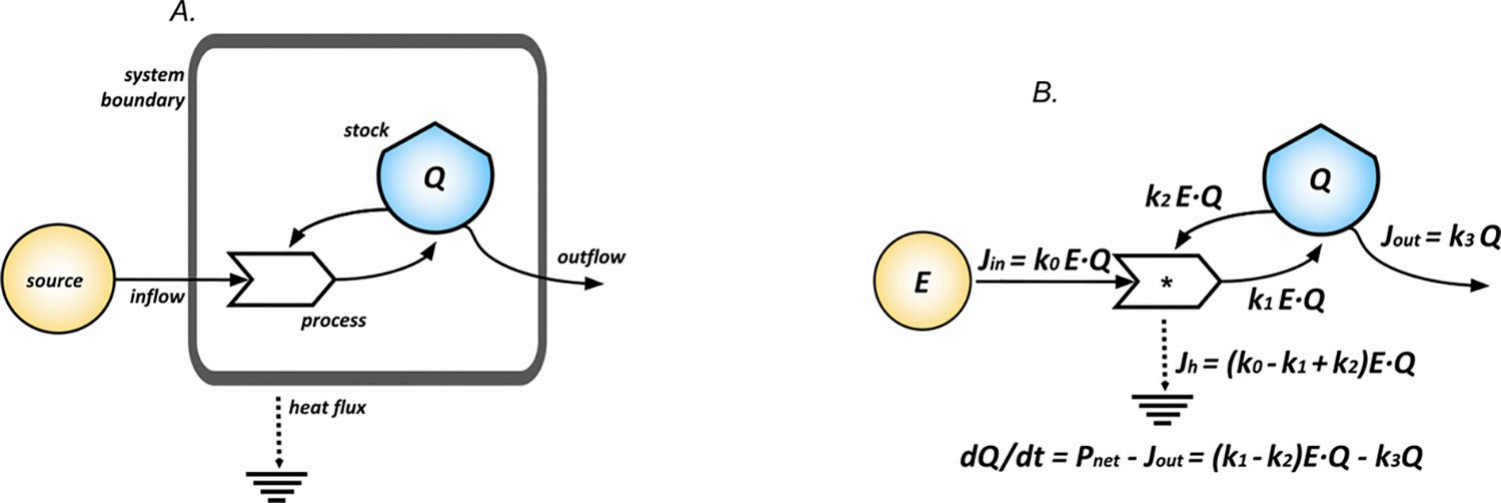
Diagram and model for autocatalytic growth. This figure shows how to derive differential equations for the evolution of the stock *Q* from the elementary diagram in A. Diagram (A) represents a system with self-reinforcing feedback. Panel (B) shows the dynamical model associated with the diagram in (A). The time evolution of the stock is modeled as a first-order time dependent differential equation for *Q*, that defines the system state at any time.

System diagrams are used to construct the model equations describing the time evolution of the stocks *Qk* (with *k = 1…N* defining their number). The diagram in Fig 6A represents a system with self-reinforcing feedback: a boundary is defined separating the external source, *E*, from the stock, *Q*, that is filled owing to the main inflow from the external source reinforced by the control flow (of energy, matter or information) from the stock. The net inflow for *Q* is given by *Pnet*. The stock is then depleted by an outflow, *Jout*, whereas the flow to the heat sink, *Jh*, represents the energy lost in the process occurring. The dynamical model associated with the diagram is given by *dQ*/*dt* = *P*_*net*_−*J*_*out*_. At the level of the process the flows are all proportional to *E·Q* because of the pairwise interaction between the source, *E*, and the stock, *Q*. The partitioning of the flows must fulfill the laws of thermodynamics and is regulated by the strength of the kinetic constants, the *k*_*s*_. Of course, the non-zero heat flow, *Jh*, is a measure of the dissipation in the process, and, in a nutshell, implements the second law of thermodynamics, whereas the balance *J*_*h*_ = *J*_*in*_−*P*_*net*_ is a consequence of the first law. The outflow is proportional to *Q* following the setting of linear irreversible thermodynamics [52,53]. The time evolution of the stock is modeled as a first-order time dependent balance equation for *Q(t)*, that defines the system state at any time *t*.

### 4.2 The self-limited autocatalytic cycle model

**Fig 7:**
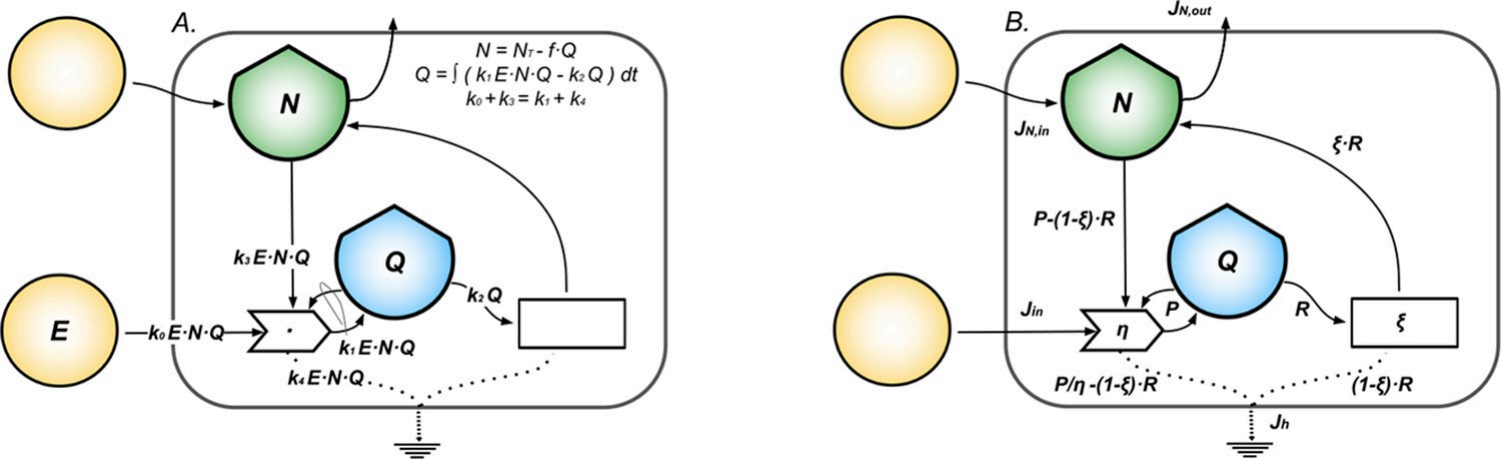
Self-limiting autocatalytic cycle model. The figure shows how to constrain and balance the stock-flow model used to describe the growth of a single cell population. Diagram (A) represents a self-limiting autocatalytic cycle with the relevant flows interacting through the production process and proportional to *E·N·Q* and the outflow proportional to *Q*. Upper right a summary of the system dynamics for the stock *N* and *Q* with the first law constraining the kinetic coefficients. Panel (B) shows the non-equilibrium thermodynamics representation for the self-limiting cycle in diagram (A). We define: the primary energy inflow, *J*_*in*_ = *k*_0_*ENQ*; the stock of limiting factors, *N*, with its inflow and outflow from the external environment *JN*,*in* = *JN*,*out*; the production and consumption processes efficiencies, *η* and *ξ*; the cells stock, *Q*, with its net power inflow, *P* = *k*_1_*ENQ* = *ηJ*_*in*_, and energy outflow, *R* = *k*_2_*Q* = *Q*/*τ*; the recycling and control feedback flows, *ξ·R* and *P*–*(1*–*ξ)·R*; the total heat flow, *J*_*h*_ = *P*/*η* = *J*_*in*_, which is the sum of the heat flows associated to the production, *P/η–(1*–*ξ)·R*, and consumption process, *(1*–*ξ)·R*. *Jh* is a measure of the irreversibility of the modeled growth process.

The dynamics of a self-regulating cell population constrained by limited resources is modeled as the self-limiting autocatalytic cycle shown in the diagram in Fig 7. In Fig 7B we explicitly show the energetic constraints obtained by balancing the energy flows through the diagram. Two stocks are involved in the system’s dynamics, *N*, the stock of resources, and *Q*, the stock of cells. The dynamics of *N* is prescribed to ensure the conservation of mass during cycling, that is constrained by the total available limited resources, *NT*:

$$N=N_{T}-f\cdot Q,$$ (Eq 8)

where *f* is defined as the fraction of *N* that *Q* stores in its structure. *N* is defined as the stock of available resources participating in the production process. In the model, *N* represents a stock of resources made of biological matter needed to produce ATP (recycled within the cell population system through self-regulation or coming from the specific micro-environment), which is represented, in an abstract way, by the physical space needed for the proliferation of the cells. A decrease of *Q* makes more space available, thus increasing *N*, even though *Q* and *N* are in this case of different nature. The replacement of a cell in *Q* with a new cell coming from the production process is from a systemic point of view a recycling of one unit of available space, going back to the *N* stock. It is worth noting how this kind of representation actually captures the self-limiting dynamic. *N* works as an internal stock that decouples the dynamics of *Q* from the external sources different from the primary (virtually unlimited) energy source, *E*. This constrains the growth of *Q*. Following the rules of energy diagrams, we define the growth equation for *Q*:

$$\frac{dQ}{dt}=P-R=k_{1}E\cdot N\cdot Q-k_{2}Q$$ (Eq 9)

were *P* = *k*_1_*E*·*N*·*Q* is the net inflow in the stock *Q* and *R* = *k*_2_*Q* is the outflow (*k1* and *k2*, are respectively the kinetic coefficients regulating the inflow and the outflow rates for *Q*). In Eq 9, we assume the production process to work as a self-reinforcing feedback for *Q* which involves the interaction between *E*, *N* and *Q* itself. The process of *Q* proliferation is a direct reinforcing feedback, playing the role of a driving force for the use of further resources and therefore entering the expression of *P*. The factors *E* and *N* represent the resources available, while the factor *Q* measures the individuals within the cell stock, whose proliferation will depend on the *Q* value independently of its role in the provision of *N*. All the flows interacting in this process are proportional to *E·N·Q* and their partitioning must be balanced. This is imposed by a condition on the kinetic constants–as shown in Fig 7A–so that *k0+k3 = k1+k4*, where *k0*, *k3* and *k4* respectively regulate the strength of the total inflow, the feedback and the heat flow. The expression *P* is at the origin of the self-limiting dynamics of *Q*. The inflow is directly proportional to *N*, which decreases as *Q* grows because of cycling for structure maintenance. This represents the fundamental feedback from which we derive the logistic growth equation for *Q* by combining Eqs 6 and 7 so that

$$\frac{dQ}{dt}=k_{1}E\cdot N_{T}\cdot Q-k_{1}fE\cdot Q^{2}-k_{2}Q$$ (Eq 10)

Stock *N* works as an overall control for the system that ensures the existence of a stable-steady state for *Q*. Moreover, it plays a major role in defining the non-linearity of the system dynamics. Using *N* as a single stock of limiting factors is an approximation based on the system description in energy units at the scale of the tissue. This is reasonable so far, we want to describe the very basic control mechanism that decouples the cellular system from the external microenvironment by providing the adaptive mechanism needed to reach a steady state through self-regulation. We reformulate Eqs 6 and 8 by redefining its parameters (28)] to obtain the classical form of the logistic growth model in Eq 1 as shown in Table 1.

**Table 1:** Relevant quantities and parametrization for the growth model of a single cell population.

Biophysical quantity	ST-based parametrization	Energetics	Parameters
ATP production flow *(P)* with resources stock *(N)*	*P = k1 E·N·Q* with *N = NT–f·Q*	*P = ηJin = rQ·(1*–*Q/K)* with *N = K–Q*	*K = NT/f (* * *) r = k1 E·NT (* ** *)*
ATP investment flow for structure maintenance *(R)*	*R = k2 Q*	*R = Q/τ*	*τ = 1/k2 (* *** *)*

*Q*, cells stock; *η*, efficiency of ATP production; *Jin*, primary energy inflow

*carrying capacity

**growth rate

***turnover time

The stability of *Q* is determined by the potential *V(Q)* defined by *dQ*/*dt* = −*dV*/*dt*, obtained by integrating Eq 1 using the physical concept of scalar potential [32,33]

$$V(Q)=\int (R-P)\cdot dQ=\frac{Q^{3}}{3\cdot K}+\frac{Q^{2}}{2\cdot \tau }-r\cdot \frac{Q^{2}}{2}$$ (Eq 11)

### 4.3 The maximum power limit

**Fig 8:**
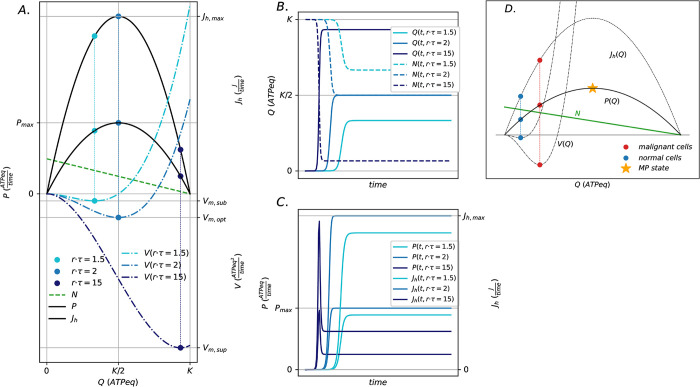
Stable stationary states and dynamics of the self-limiting autocatalytic cycle. Panel (A) shows the model state space defined by the curves for the power (bold black), *P*(*Q*) = *r*·*Q*·(1−*Q*/*K*), the heat flow (black), *J*_*h*_(*Q*) = *P*(*Q*)/*η* and the stability potential (dot dashed), *V*(*Q*) = *a*·*Q*^3^+*b*·*Q*^2^ (coefficients in Eq 8). The colored dots on the curves represent the steady-states for *P*, *Jh* and *V* as a function of *Q* (*0*<*Q<K*) for the three possible different growth regimes, respectively, defined by the product *r·τ*: sub-optimal, *r·τ = 1*.*5* (azure), optimal or maximum power state (*Pmax*), *r·τ = 2* (blue), and super-optimal, *r·τ = 10* (dark-blue). This is associated with the stock *N = K-Q* (dashed lines) that decreases for increasing *Q* to fulfill the mass balance constraint. Panels (B) and (C) show the time evolution associated with the configurations defined by the three different growth regimes (azure, blue, dark blue), respectively, for the stocks *Q* and *N* (dashed), and for the flows *P* and *Jh*. Panel (D) shows the application of the formulation to estimate the stationary states of normal and neoplastic plasma cells.

The maximum power state generally represents one of the possible steady states that can be reached by a growing population under such conditions, and it can be a transient state for populations growing up to the carrying capacity. It represents the state in which the primary energy inflow is converted into free energy (ATP) at the maximum possible rate, establishing a limit to energy conversion as shown in Fig 8. The existence of a maximum power (MP) limit in the system is a consequence of resource limitation under mass balance constraint. The energy stored into the stock *Q* is used by the cells stock to perform work. Part of it is used to grow in volume and duplicate, hence for proliferation, parametrized and estimated by the growth rate, *r*. Part of it is used to develop feedback on the production process by means of biosynthesis on a typical timescale, *τ*, we estimate as the proteome turnover time. The latter defines the flow of energy that is used to maintain the living structure, *R = Q/τ*, as work per unit time. *R* measures part of the energy flow used by cells to counteract the degradation of the living structure. The parameter *ξ* represents the fraction of *R* (*0<ξ<1*) that is recycled within the system, *ξ·R*, and feeds back into the stock *N*, by directly regulating the dissipation flow due to biosynthesis. Conservation of mass is imposed by Eq 8. This control feedback represents the overall system regulation mechanism that allows the whole system to function and survival. The recycling flow is associated with the chemical energy stored in functional biomolecules, enzymes, molecular machines, energetic molecules and other biosynthesis products that restore the membrane potentials and chemical potential gradients that drive, fuel and allow cellular respiration pathways and control the interaction of cells with the external environment. In this sense, it is reasonable to use a single stock, although it is a first order approximation.

The analytical expression for *P* is quadratic and admits a unique maximum that we define as the maximum power state (MP state). This state is a benchmark for the evolution of the whole system as a function of the parameters *r*, *τ* and *K*. The following analytical expressions are obtained from Eq 1 for *Q*, *P* and *Jh* in steady state:

$$Q_{ss}=K\cdot (1-\frac{1}{r\cdot \tau })$$ (Eq 12)

$$P_{ss}=\frac{K}{\tau }\cdot (1-\frac{1}{r\cdot \tau })$$ (Eq 13)

$$J_{h,ss}=\frac{K}{\eta \cdot \tau }\cdot (1-\frac{1}{r\cdot \tau })$$ (Eq 14)

The steady states in Eqs 12, 13 and 14 reflect open boundary conditions, as shown in Fig 2. For fixed *K*, the quantity *r·τ* controls the steady states, parametrizing a minimal *rK*-selection mechanism characterized by the following growth regimes:

- a sub-optimal regime for *1 < r·τ < 2* with logistic growth of *Q*, *P* and *Jh* (Fig 8, in azure) approaching stable steady states characterized by *0 < Qss < K/2*, *0 < Pss < Pmax* and *0 < Jh*,*ss < Jh*,*max*;
- an optimal regime for *r·τ = 2*, defined as the maximum power state, with logistic growth for *Q*, *P* and *Jh* (Fig 8, in blue) approaching a unique stable steady state characterized by *Qss*,*opt = Nss*,*opt = K/2*, *Pss = Pmax = K·r/4* and *Jh*,*ss = Jh*,*max = Pmax/η*;
- a super-optimal regime for *r·τ > 2* with logistic growth for *Q*, while *P* and *Jh* undergo an overshoot dynamics (Fig 8, in dark blue) reaching stable-steady states characterized by *K/2 < Qss < K*, *0 < Pss < Pmax* and *0 < Jh*,*ss < Jh*,*max*;

The growth of *Q* increases the *P* inflow while depleting the stock *N*. This dynamic generates a trade-off between *P* and *N* from which the maximum power state emerges. The quadratic expression for *P* as a function of *Q* (Table 1) reflects this limitation. In terms of the stability, the higher *r·τ*, the more the potential well defined by *Vm* deepens (Fig 8A, dot dashed lines), the more stable the system is.

### 4.4 The competition model

**Fig 9:**
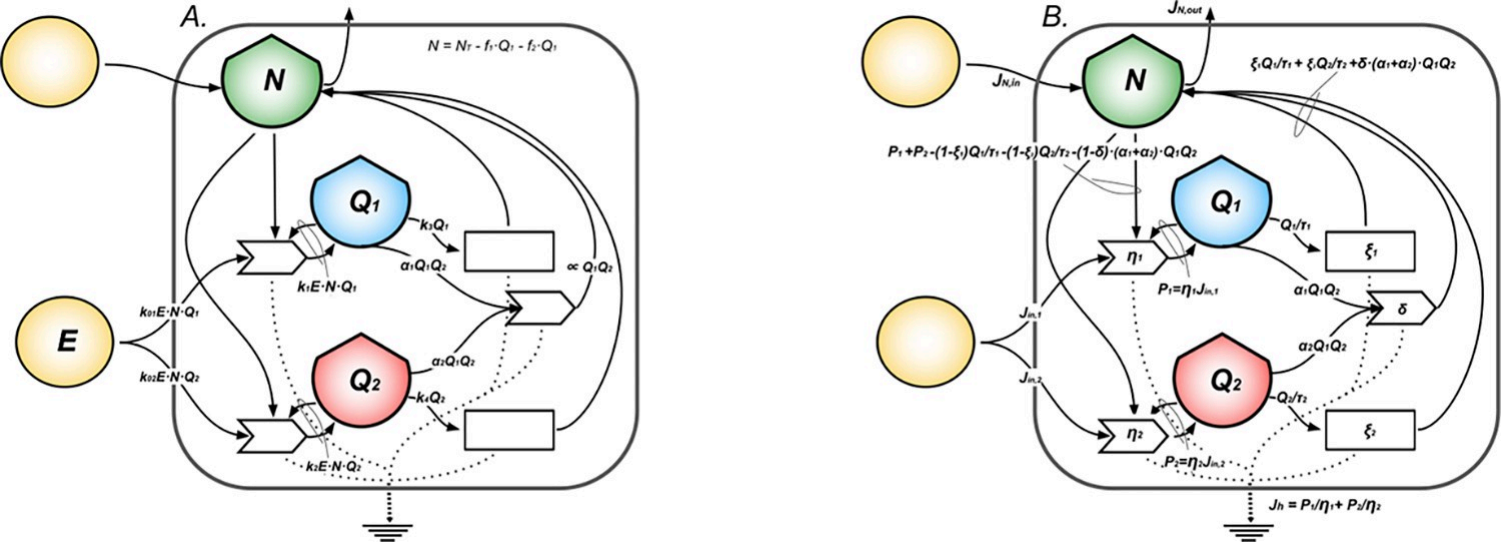
Competition dynamics and energetic constraints. This figure shows how to constrain and balance the stock-flow model used to describe the growth of interacting cell populations. Both (A) and (B) represent a system of stocks competing for space. In diagram (A), the upper right shows the mass balance constraint on *N*, with power inflows proportional to *ENQ1* or *Q2* and respectively with outflows proportional to *Q1* or *Q2*. The interaction term is a Lotka-Volterra-like proportional to *Q1·Q2*. The primary energy inflow, *J*_*in*_ = *k*_0,1_*ENQ*_1_+*k*_0,2_*ENQ*_2_ is transformed into useful power flows *P*_1_ = *k*_1_*ENQ*_1_ = *η*_1_*J*_*in*,1_ and *P*_2_ = *k*_2_*ENQ*_2_ = *η*_2_*J*_*in*,2_ for the two stocks with efficiencies, *η1* and *η2*; The efficiencies *ξ1* and *ξ2* control the partitioning energy outflows *R*_1_ = *k*_3_*Q*_1_ = *Q*_1_/*τ*_1_; and *R*_2_ = *k*_4_*Q*_2_ = *Q*_2_/*τ*_2_ between recycling feedback flows and the heat flows. The total heat flow *J*_*h*_ = *J*_*in*_ = *P*_1_/*η*_1_+*P*_2_/*η*_2_ sinks out of the boundary at the bottom of the diagram, with 0<*δ<1* being the phenomenological efficiency for the direct interaction term.

We define the competition model extending the self-limited autocatalytic cycle model for two interacting stocks competing for limited resources. Three stocks are involved in the system dynamics: two population stocks, *Q1* and *Q2*, and the stock of the limited available resources, *N*. The model is built upon the self-limiting growth archetype in Eq 9 as follows:

$$\frac{dQ_{1}}{dt}=P_{1}-R_{1}-I_{1}=k_{1}ENQ_{1}-k_{3}Q_{1}-\alpha_{1}Q_{1}\cdot Q_{2}$$ (Eq 15)

$$\frac{dQ_{2}}{dt}=P_{2}-R_{2}-I_{2}=k_{2}ENQ_{2}-k_{4}Q_{2}-\alpha_{2}Q_{1}\cdot Q_{2}$$ (Eq 16)

$$N=N_{T}-f_{1}Q_{1}-f_{2}Q_{2}$$ (Eq 17)

were *P*_1_ = *k*_1_*ENQ*_1_ and *P*_2_ = *k*_2_*ENQ*_2_ are the net inflows in the stock *Q1* and *Q2*, respectively, with outflows *R*_1_ = *k*_3_*Q*_1_ and *R*_2_ = *k*_4_*Q*_2_ (*k1* and *k2*, are the kinetic coefficients regulating the inflow rates in *Q1* and *Q2*, *k3* and *k4* regulates the correspondent outflow rates). The terms *I*_1_ = *α*_1_*Q*_1_*Q*_2_ and *I*_2_ = *α*_2_*Q*_1_*Q*_2_ represent the biochemical interaction between the populations. Both terms have the structure of Lotka-Volterra interactions with strengths *α1* and *α2*. As for Eq 9, we assume the production processes to work as a self-reinforcing feedback for both *Q1* and *Q2*, involving the interaction between *E*, *N* and *Q1* or *Q2*, as shown in Fig 9. For competing populations, the resource dynamics (Eq 17) changes, with respect to Eq 8, as a consequence of competition for space. The total available resources *NT* are reduced by the growth of both *Q1* and *Q2*, that incorporate respectively a constant fraction of nutrients *f1* and *f2* within them. This results in the reduction of the net inflows *P1* and *P2* by an additional term proportional to *Q1*·*Q2* that parametrizes competition for space in both Eqs 15 and 16. These interaction terms are also Lotka-Volterra-like. We redefine the parameters in Eqs 15–17 as for Eqs 8 and 10, obtaining Eqs 3 and 4 (see Table 2 for details).

**Table 2:** Biophysical quantities and parameterization for competing cell populations.

Biophysical quantity	ST-based parameterization	Energetics	Parameters
ATP production flow *(P1*,*P2)* with resources stock *(N)*	*P1 = k1 E·N·Q1* *P2 = k2 E·N·Q2* with *N = NT–f1·Q1- f2·Q2*	*P1 = η1Jin*,*1 = r1Q1·(1-Q1/K*–*Q2/K)* *P2 = η2Jin*,*2 = r2Q2·(1-Q1/K*–*Q2/K)* with *N = K - Q1- Q2*	*K = NT/f1 = NT/f2 (* * *)* *r1 = k1 E·NT* *r2 = k2 E·NT (* ** *)*
ATP investment flow for structure maintenance *(R1*,*R2)*	*R1 = k3 Q1* *R2 = k4 Q2*	*R1 = Q1/τ1* *R2 = Q2/τ2*	*τ1 = 1/k3* *τ2 = 1/k4 (* *** *)*
Biochemical interaction (*I1*,*I2*)	*I1 = α1Q1Q2* *I2 = α2Q1Q2*		

*Q1* and *Q2*, competing cells stock; *η1* and *η2*, efficiencies of ATP production; *Jin = Jin*,*1+Jin*,*2*, primary energy inflow; *α1* and *α2*, strength of apoptosis control

*carrying capacity

**growth rates

***turnover times

The stability to small perturbations of the stationary states of Eqs 3 and 4 given by Eqs 5 and 6 is determined from the sign of the eigenvalues λ obtained from the characteristic equation (see [54] for the original study):

$$\left|\begin{array}{cc} (r_{2}-\frac{1}{\tau_{2}})-K\cdot (1-\frac{1}{r_{1}\tau_{1}})\cdot (\frac{r_{2}}{K}+\alpha_{2})-\lambda & 0\\ -K\cdot (1-\frac{1}{r_{1}\tau_{1}})\cdot (\frac{r_{1}}{K}+\alpha_{1}) & -(r_{1}-\frac{1}{\tau_{1}})-\lambda \end{array}\right|=0$$ (Eq 18)

which yields the following condition for the stability of *(a)* in Eq 5:

$$\alpha_{2}>\frac{r_{2}}{K}\left[\frac{\left(1-\frac{1}{r_{2}\tau_{2}}\right)}{\left(1-\frac{1}{r_{1}\tau_{1}}\right)}-1\right]$$ (Eq 19)

that never holds for the parameter estimation shown in Table 3 and the simulations shown in Fig 5. Thus, ${Q_{1,ss}}^{\left(a\right)}=K\cdot (1-\frac{1}{r_{1}\cdot \tau_{1}}){Q_{2,ss}}^{\left(a\right)}=0$ is an unstable stationary state, while ${Q_{2,ss}}^{\left(b\right)}=K\cdot (1-\frac{1}{r_{2}\cdot \tau_{2}}){Q_{1,ss}}^{\left(b\right)}=0$ is stable, being $\alpha_{2}>\frac{r_{2}}{K}\left[\frac{\left(1-\frac{1}{r_{2}\tau_{2}}\right)}{\left(1-\frac{1}{r_{1}\tau_{1}}\right)}-1\right]$ always verified in our setting.

**Table 3:** Parameter setting.

parameter	estimated value	reference
*K*, carrying capacity*	*4*.*05·10*^*21*^ *ATPeq*	[55,59]
*Qss*, normal PCs steady-state**	*8*.*1·10*^*19*^ *ATPeq*	[55,59,60]
*η*, efficiency of ATP production (*η*_*1*_ *= η*_*2*_)	*40%*	[61] (ox.-phos. pathway)
*τ*, proteome turnover time for normal and malignant PCs (*τ*_*1*_ *= τ*_*2*_ in Eqs10-11)	*72 h*	[36,62–64]
*r*_*m*_, growth rate for malignant PCs*** (*r*_*2*_ in Eqs10-11)	1.43·10^−2^*h*^−1^−1.53·10^−2^*h*^−1^	[65–68]
*r*_*n*_, growth rate for normal PCs**** (*r*_*1*_ in Eqs10-11)	1.39·10^−2^*h*^−1^	this work
*α1* and *α2*, strength of apoptosis control*****	10^−18^*h*^−1^*ATPeq*^−1^	this work

* *K≅*(n. of cells in human body)·(% bone marrow volume in human body)·(ATP in a cell)

** *Qss ≅ 0*.*02·K*

*** r_*m*_ *= RGR + 1/τ* with *RGR =* 3%-4%-6%-10%

where $\frac{dQ}{dt}≃RGR\cdot Q=(r-\frac{1}{\tau })\cdot Q$ small time approximation of Eq 6 resampling ideal in-vitro experiments

**** r_*n*_ *≅ 1/[τ·(1-Qss/K)]*

***** *α1 = c·r1*/*K* and *α2 = c·r2*/*K* with *c = 0*,*1*,*2*

### 4.5 Parameter estimation and sensitivity analysis

Stocks are measured in ATP equivalent units. The procedure consists in converting cell stocks into equivalent ATP stocks. The number of cells in a stock is converted to the correspondent number of ATP molecules stored in it. The equivalence is based on the estimation provided in [55]. The number of ATP molecules to build up a single cell is *5·10*^*10*^ *ATP*, hence *1 cell~5·10*^*10*^ *ATPeq*. In joules, 1*ATP*10^−19^ joules [56], thus 1*cell*5·10^10^*ATPeq*5·10^−9^ joules.

We estimate the parameters in Eqs 1, 3 and 4 to obtain plausible scenarios for the time evolution of the cell population systems. As an application of our modeling scheme, we calibrate the models based on biophysical and biomedical literature concerning human plasma-cells (PCs) and malignant plasma-cells. Table 3 summarizes the parameter setting, calculation methods and the literature. More details about the calculations are available in Section A in S1 Appendix.

Numerical sensitivity analysis consists in varying the values of one parameter at the time (OAT) by keeping the others fixed while running a simulation. We perform a numerical sensitivity analysis for the parameter *r2*, in Eq 4. We fix *τ2 = 72 h* and vary *r2* within the typical range estimated for neoplastic PCs (Table 3). The corresponding analytical sensitivities for the stock (*Q*) and the power input (*P*) are computed from the single population model in Section B in S1 Appendix.

**Fig 10:**
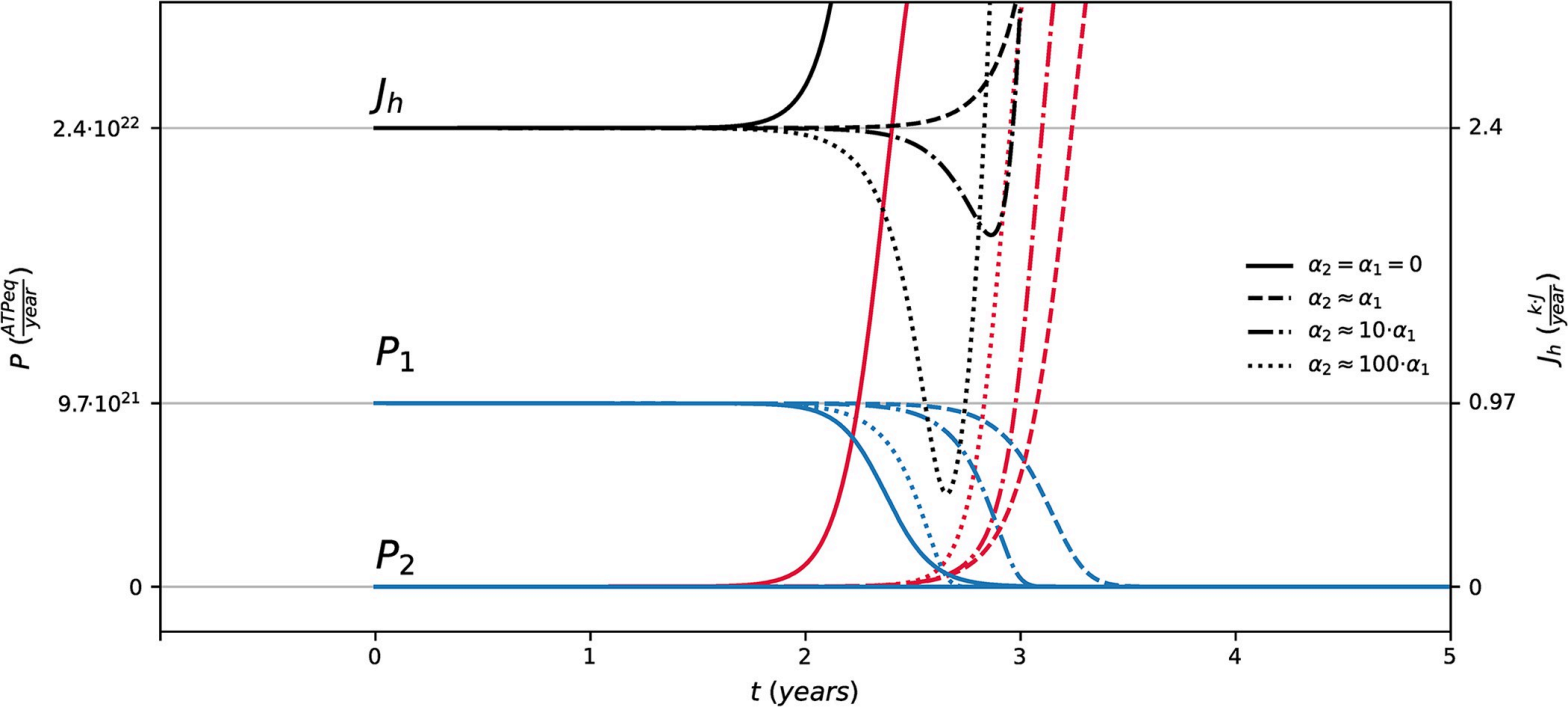
Simulation of the power flows and the total heat flow for competing cells stocks. It shows the time evolution on the yearly time scale for the power flows *P1* (blue) and *P2* (red), and the total heat flow *Jh* (black), for the different values of *α1/α2* represented by the different line styles, as in the legend. The setting for the other parameters follows the values reported in Table 3: *K = 4*.*05·10*^*21*^ *ATPeq*, *Q*_*1*,*0*_ *= 8*.*1·10*^*19*^ *ATPeq*, *Q*_*2*,*0*_ *= 5·10*^*10*^ *ATPeq*, *η = 40%*, *τ*_*1*_ *= τ*_*2*_ *= 72 h*, *r*_1_ = 1.39·10^−2^*h*^−1^, *r*_2_ = 1.53·10^−2^*h*^−1^.

We run a second numerical sensitivity analysis by changing the strength of the biochemical interactions *α1* and *α2* in Eqs 3 and 4. We keep their ratio constant to *α1*/*α2 = r1*/*r2* without changing their order of magnitude, so that *α1 = c·r1*/*K* and *α2 = c·r2*/*K* with *c = 0*, *1*, *2*. This choice reflects the ability of cells to perform physical work, parametrized through the production rates *r1* and *r2*. As an example of possible scenarios that can be constructed from Eqs 3 and 4, Fig 10 shows the patterns obtained by exploring the parameter space of the ratio *α1/α2* by fixing all the other parameters in the equations. The figure shows the time evolution of the power flows *P1* and *P2* and of the total heat flow *Jh*.

### 4.6 Initial conditions and integration scheme

The simulation study concerns the time integration of Eqs 3 and 4 for specific model configurations defined by the parameters estimated in Table 3 and the initial conditions defined in this section. As an integration scheme, we use a numerical implicit Runge-Kutta integration method known as Radau method IIA [57]. The code is implemented in Python language (available in the Section C in S1 Appendix) and the solver is available from Python numerical methods library [58]. The integration time step is set to be *Δt = 0*.*01 years* as we aim at studying the system dynamics on timescales comparable with the human lifespan. The initial condition for the stock of malignant cells is set to 1 cell in ATP equivalent units, thus *Q(0) = 5·10*^*10*^ *ATPeq*. In the competition scheme defined by Eqs 3–4 the initial conditions are set in a way that the initial presence of the neoplastic plasma-cells results as the smallest perturbation possible for the adapted population of normal PCs in steady-state. Thus, *Q1(0) = Qss = 8*.*1·10*^*19*^ *ATPeq*, for the stock of normal plasma cells initially in steady-state, and *Q2(0) = 5·10*^*10*^ *ATPeq*, equivalent to 1 mutated (malignant) plasma cell in the bone marrow.

## Supporting information

S1 Appendix Section A: Parameter estimation. Section B: Analytical sensitivity analysis of the single population model. Section C: Simulator of the interacting population model as Python routine.
(PDF)
