# Supplementary material for: Modeling cell populations metabolism and competition under maximum power constraints
Source: PLoS Comput Biol. 2023 Nov 8;19(11):e1011607. doi: 10.1371/journal.pcbi.1011607 (PMC10659174; doi:10.1371/journal.pcbi.1011607)
Supplement: S1 Appendix — Section A: Parameter estimation. Section B: Analytical sensitivity analysis of the single population model. Section C: Simulator of the interacting population model as Python routine. (PDF) [file pcbi.1011607.s001.pdf]

Supporting Information

for the article ***Modeling cell populations metabolism and competition under maximum power constraints*** by Conte L. et al.

A. Parameter estimation

We consider the bone marrow (BM) as the tissue where normal and malignant PCs grow - playing the role of the micro-environment. We estimate the carrying capacity K as the total number of cells that can proliferate in the bone marrow in ATP equivalent units so that $K \sim 4.05 \cdot 10^{21}$ *ATPeq* with

$$K \cong (\text{n. of cells in human body}) \cdot (\text{\% bone marrow volume in body}) \cdot (\text{ATP in a cell}) \qquad \text{(S1)}$$

where the total number of cells in the human body is $3 \cdot 10^{12}$ cells [1; 2], the volume occupied by the bone marrow (1.75 L) [1] within a standard weight human body (65 L) [1] is ~2.7% of the total body volume, and the ATP required to build up a cell is $5 \cdot 10^{10}$ ATP molecules per cell [1; 2].

We estimate the steady-state value of the stock of the adapted population (normal PCs), Q_{ss}, as the average fraction of plasma-cells typically found in the BM, so that $Q_{ss} \sim 8.1 \cdot 10^{19}$ *ATPeq* $\cong 0.02 \cdot K$, where the average fraction of plasma cells in the bone marrow is ~2% [3].

The phenomenological efficiency η is estimated for both normal and neoplastic plasma cells ($\eta_1 = \eta_2$) as the efficiency of mitochondrial respiration $\eta \sim 40\%$ [4].

We estimate the parameter τ from measures of cell proteome turnover time, defined as the time for a cell to completely regenerate its proteome with newly synthesized proteins. From quantitative proteomics studies - performed with stable isotope labeling by amino acids in cell culture (SILAC) techniques [5; 6; 7; 8] we estimate $\tau \sim 72\ h$ both for normal and neoplastic PCs.

We estimate the parameter r for malignant PCs (r_m) from observations of proliferation rates of neoplastic plasma cell populations combined with the estimate for $\tau \sim 72\ h$. The proliferation rate, or relative growth rate (*RGR*), is typically measured with the Ki-67 flow cytometry technique performed on clinical samples for patients affected by multiple-myeloma disease at different developmental stages [9; 10; 11; 12]. We estimate r_m from the approximation of Eq.5 for small times, resampling in-vitro experimental conditions. For small times, Eq.5 has an exponential solution for Q that only depends on the relative growth rate $RGR = r - 1/\tau$

$$\frac{dQ}{dt} \simeq RGR \cdot Q = \left(r - \frac{1}{\tau}\right) \cdot Q \qquad \text{(S2)}$$

thus $r = RGR + 1/\tau$. We us1 reported typical values for *RGR* (3%-10%) and τ (72 h) to estimate $r_m \sim 1.43 \cdot 10^{-2}$ - $1.53 \cdot 10^{-2}\ h^{-1}$ for different malignant PCs phenotypes.

We infer the parameter r for normal PCs (r_n) inverting the analytical expression for $Q_{ss} = K \cdot (1 - 1/r \cdot \tau)$, from estimates of $Q_{ss} \sim 8.1 \cdot 10^{19}$ *ATPeq*, $K \sim 4.05 \cdot 10^{21}$ *ATPeq* and $\tau \sim 72\ h$. Thus $r_n \sim 1.39 \cdot 10^{-2}\ h^{-1}$ for normal PCs.

B. Analytical sensitivity analysis

We derive the analytical sensitivities of the quantities *Q* and *P* in steady state, Q_{ss} and P_{ss}, for the single population model in Eq. 7. The expression for $Q_{ss}=K\cdot(1-1/r\cdot\tau)$ and $P_{ss}=(K/\tau)\cdot(1-1/r\cdot\tau)$ both depend on *K*, *r* and *τ* . Their sensitivity with respect to change in these parameters is calculated as differentials of thermodynamic observables

$$dP_{ss}=\left(\frac{\partial P_{ss}}{\partial K}\right)_{r,\tau}dK+\left(\frac{\partial P_{ss}}{\partial r}\right)_{K,\tau}dr+\left(\frac{\partial P_{ss}}{\partial \tau}\right)_{K,r}d\tau \qquad \text{(S3)}$$

$$dQ_{ss}=\left(\frac{\partial Q_{ss}}{\partial K}\right)_{r,\tau}dK+\left(\frac{\partial Q_{ss}}{\partial r}\right)_{K,\tau}dr+\left(\frac{\partial Q_{ss}}{\partial \tau}\right)_{K,r}d\tau \qquad \text{(S4)}$$

In our case study, we fix *τ* and *K* and change *r* for different phenotypes of neoplastic plasma cells, exploiting the approximation for small times described in Section A of S1 Appendix. Thus - keeping $dK=0$ and $d\tau=0$ - the sensitivity of Q_{ss} and P_{ss} to changes in *r* - with respect to the reference normal state r_n - is given by

$$dP_{ss}=\frac{K}{{r_n}^2\cdot\tau^2}\cdot dr\sim 4\cdot10^{21}ATPeq\cdot dr \qquad \text{(S5)}$$

$$dQ_{ss}=\frac{K}{{r_n}^2\cdot\tau}\cdot dr\sim 3\cdot10^{23}ATPeqh^{-1}\cdot dr \qquad \text{(S6)}$$

The actual change in P_{ss} and Q_{ss} can be inferred from Eqs. S5 and S6 with respect to the change in *r* (*dr*) for increasing malignancy of the neoplastic plasma cells (r_m) with respect to normal PCs (r_n) - as shown in the table below (and Fig 4).

Table A. Analytical sensitivity analysis for the single population model applied to PCs

$dr=r_m-r_n$	$0.04\cdot10^{-2}\,h^{-1}$	$0.05\cdot10^{-2}\,h^{-1}$	$0.08\cdot10^{-2}\,h^{-1}$	$0.14\cdot10^{-2}\,h^{-1}$
dP_{ss}	$0.16\cdot10^{19}ATPeq\,h^{-1}$ ~ $0.03\cdot K/\tau$	$0.20\cdot10^{19}ATPeq\,h^{-1}$ ~ $0.04\cdot K/\tau$	$0.32\cdot10^{19}ATPeq\,h^{-1}$ ~ $0.06\cdot K/\tau$	$0.56\cdot10^{19}ATPeq\,h^{-1}$ ~ $0.1\cdot K/\tau$
dQ_{ss}	$0.12\cdot10^{21}ATPeq$ ~ $0.03\cdot K$	$0.15\cdot10^{21}ATPeq$ ~ $0.04\cdot K$	$0.24\cdot10^{21}ATPeq$ ~ $0.06\cdot K$	$0.42\cdot10^{21}ATPeq$ ~ $0.1\cdot K$

C. Simulator as Python routine

The routine can be run as .py script once included the following libraries in a Python environment:

- numpy (https://numpy.org/doc/stable/user/index.html#user)
- matplotlib (https://matplotlib.org/stable/index.html)
- scipy (https://docs.scipy.org/doc/).

The actual parameter setting and aesthetics reproduces Fig 10 of the main text. It is straightforward to extend the method for the numerical sensitivity analysis to other model parameters.

```
#!/usr/bin/env python3
# -*- coding: utf-8 -*-

#importing python libraries
import numpy as np
import matplotlib.pyplot as plt
from scipy.integrate import solve_ivp #method from scipy

#MODEL for two interacting stocks
def model_metabolism_interacting(t, z, K, r1, r2, T1, T2, a1, a2, f):

        Q1, Q2 = z #normal cells, cancer cells (first derivative)

        return [ r1*Q1*(1-Q1/K-f*Q2/K)-Q1/T1-a1*Q1*Q2,
        r2*Q2*(1-f*Q2/K-Q1/K)-Q2/T2-a2*Q1*Q2]

#SENSITIVITY to competition parameters AND REGIME SHIFTS
#SCENARIOS and model calibration
K=4.05e21                       #carrying capacity in ATP equiv , total cells in the niche
tau = 72/8760                   #TURNOVER TIME FOR BIOSYNTHESIS same for both NORMAL AND CANCER: proteome turnover
time, biochemical constraint
T1 = T2 = tau
"""
#SENSITIVITY to changes in the growth rate of one population
#sensitivity analysis on neoplastic plasma cells phenotype spans biomedical observed value (REFs)
RGR2_sens = np.array([0.03, 0.04, 0.06, 0.07, 0.08, 0.1])*(8760/72)  #%/ANNO ,0.5,0.8,0.9
r2_sens=RGR2+1/T2
"""
#malignant plasmacells
RGR2 = 0.1/T2
r2 = RGR2 + 1/T2
#normal plasmacells
Q1ss_est=8e19                   #(ATPeq) steady state normal PCs
r1 =1/(T1*(1-((Q1ss_est)/K)))  #intrinsic growth rate estimation
#RGR_1 = r1-1/T1                #correspondent RGR?
Q10 = Q1ss_est
eta = 0.4                       #thermodynamic efficiency of ATP production process: FIXED BUILDING BLOCK OF LIFE, from Stat
Mechanics
#SOLVER
Q10=K*(1-1/(r1*T1))
Q20=5e10                        #(ATPeq) first mutation, 1 cell in ATPeq
DT=80
#sensitivity to biochemical interaction strength , same order of magnitude to estimated from maximum power state Q1ss=Q2ss=K/2
a1_sens=np.array([0,1,10,100])*r1/K
a2_sens=np.array([0,1,1,1])*r2/K        #,1,10,100 #1,2,5
f=1                             #mass/material balance closure, conservative scenario, for f<1 PCs abrogate hierarchical control :
exceeds in the carrying capacity
t = np.linspace(0, DT, 100*DT)
Q1_sens = list(np.zeros(len(a1_sens)))  #change to RGR2_sens
Q2_sens = list(np.zeros(len(a2_sens)))
P1_sens = list(np.zeros(len(a1_sens)))
P2_sens = list(np.zeros(len(a2_sens)))
Jh_sens = list(np.zeros(len(a2_sens)))
```

```
for i in range(0,len(a2_sens)):
        sol_metabolismINT = solve_ivp(model_metabolism_interacting,

                [0, DT],                #timespan

                [Q10, Q20],         #initial stock condition

                t_eval = t,         #impose the timestamp

                method='Radau',     #integration method: Radau

                args=(K, r1, r2, T1, T2, a1_sens[i], a2_sens[i], f), #definition of the constants

                dense_output=True)          #True = computes continuous solution
  #solutions
        Q1_sens[i]=sol_metabolismINT.sol(t)[0]
        Q2_sens[i]=sol_metabolismINT.sol(t)[1]
        P1_sens[i]=r1*Q1_sens[i]*(1-Q2_sens[i]/K-f*Q2_sens[i]/K)
        P2_sens[i]=r2*Q2_sens[i]*(1-Q1_sens[i]/K-f*Q2_sens[i]/K)
        """
        #sensitivity to phenotype, substitute
        #P2_sens[i]=r2_sens[i]*Q2_sens[i]*(1-Q1_sens[i]/K-f*Q2_sens[i]/K)
        """
        Jh_sens[i]=P1_sens[i]/eta + P2_sens[i]/eta

#P1, P2, power in ATPeq/year - Jh, heat flows kJ/year
#DYNAMICS
fig, ax = plt.subplots(figsize=(9,4.5),dpi=400)#,dpi=400
#power flows
ax.plot(t,P2_sens[3], color="crimson",ls=":")
ax.plot(t,P1_sens[3], color="tab:blue",ls=":")
ax.plot(t,P2_sens[2], color="crimson",ls="-.")
ax.plot(t,P1_sens[2], color="tab:blue",ls="-.")
ax.plot(t,P2_sens[1], color="crimson",ls="--")
ax.plot(t,P1_sens[1], color="tab:blue",ls="--")
ax.plot(t,P2_sens[0], color="crimson")
ax.plot(t,P1_sens[0], color="tab:blue")
ax.set_ylim(-0.1*Q1ss_est/(eta*T1),Q1ss_est/(eta*T1)+0.3*Q1ss_est/(eta*T1))#+0.1*Q1ss_est/T1
ax.set_xlim(-1,5)
#heat flows
ax2 = ax.twinx()
ax2.plot(t,Jh_sens[0], color="k")
ax2.plot(t,Jh_sens[1], color="k",ls="--")
ax2.plot(t,Jh_sens[2], color="k",ls="-.")
ax2.plot(t,Jh_sens[3], color="k",ls=":")
ax2.set_ylim(-0.1*Q1ss_est/(eta*T1),Q1ss_est/(eta*T1)+0.3*Q1ss_est/(eta*T1))#+0.1*Q1ss_est/T1
ax2.set_yticklabels([r"0",r" ",r"$0.97$",r"$2.4$"],fontsize="small")
ax2.set_ylabel(r'$J_h$ $(\frac{k·J}{year})$')
ax2.set_yticks(np.array([0, P2_sens[0][0], P1_sens[0][0],Jh_sens[0][0]]))#$5·10^{10}$
#figure aestethics
ax.set_yticks(np.array([0, P2_sens[0][0], P1_sens[0][0],Jh_sens[0][0]]))#$5·10^{10}$
ax.set_yticklabels([r"0",r" ",r"$9.7·10^{21}$",r"$2.4·10^{22}$"],fontsize="small")
ax.grid(True,axis="y")
ax.set_ylabel(r'$P$ $(\frac{ATPeq}{year})$')
ax.set_xlabel(r'$t$ $(years)$')
ax.text(0,2.6e22,r"$J_h$",fontsize=15)
ax.text(0,1.2e22,r"$P_1$",fontsize=15)
ax.text(0,1e21,r"$P_2$",fontsize=15)
ax2.legend([r"$\alpha_2=\alpha_1=0$",r"$\alpha_2 \approx \alpha_1$", r"$\alpha_2 \approx 10·\alpha_1$", r"$\alpha_2 \approx
100·\alpha_1$"], shadow=False, loc="center right", fontsize="small",framealpha=0) #,"","",""
ax.set_xticklabels([r" ",r"$0$",r"$1$",r"$2$",r"$3$",r"$4$",r"$5$"],fontsize="small")

###
x=3
t1 = Q1_sens[x]/(P1_sens[x]-Q1_sens[x]/T1-a1_sens[x]*Q1_sens[x]*Q2_sens[x])
plt.plot(t,t1)
plt.xlim(-1,5)
```

References

1. Herman IP. Physics of the Human Body. 2nd ed. Cham: Springer International Publishing; 2016.
2. Milo R, Phillips R. Cell Biology by the Numbers. Garland Science; 2015.
3. SANDKÜHLER S, GROSS E. Normal Bone Marrow Total Cell and Differential Values by Quantitative Analysis of Particle Smears. Blood. 1956 Sep 1;11(9):856–62.
4. Nath S. The thermodynamic efficiency of ATP synthesis in oxidative phosphorylation. Biophys Chem. 2016 Dec;219:69–74.
5. Zatula A, Dikic A, Mulder C, Sharma A, Vågbø CB, Sousa MML, et al. Proteome alterations associated with transformation of multiple myeloma to secondary plasma cell leukemia. Oncotarget. 2017 Mar 21;8(12):19427–42.
6. Pino LK, Baeza J, Lauman R, Schilling B, Garcia BA. Improved SILAC Quantification with Data-Independent Acquisition to Investigate Bortezomib-Induced Protein Degradation. J Proteome Res. 2021 Apr 2;20(4):1918–27.
7. Liu TY, Huang HH, Wheeler D, Xu Y, Wells JA, Song YS, et al. Time-Resolved Proteomics Extends Ribosome Profiling-Based Measurements of Protein Synthesis Dynamics. Cell Syst. 2017 Jun;4(6):636-644.e9.
8. Cambridge SB, Gnad F, Nguyen C, Bermejo JL, Krüger M, Mann M. Systems-wide Proteomic Analysis in Mammalian Cells Reveals Conserved, Functional Protein Turnover. J Proteome Res. 2011 Dec 2;10(12):5275–84.
9. Juskevicius R, Murthy H, Dangott B. Plasma Cell Myeloma With Very High Ki67 Proliferation Rate: Comparison of Visual Estimation and Computational Image Analysis With Description of Clinical and Pathologic Features. Am J Clin Pathol. 2015 Oct 1;144(suppl 2):A132–A132.
10. Gastinne T, Leleu X, Duhamel A, Moreau A, Franck G, Andrieux J, et al. Plasma cell growth fraction using Ki-67 antigen expression identifies a subgroup of multiple myeloma patients displaying short survival within the ISS stage I. Eur J Haematol. 2007 Oct 10;79(4):297–304.
11. Markovic O, Marisavljevic D, Cemerikic V, Vidovic A, Bakrac M, Perunicic M, et al. Proliferative activity of myeloma cells determined by Ki-67 antibody: Biological and clinical significance. Vojnosanit Pregl. 2005;62(1):33–8.
12. Alexandrakis MG, Passam FH, Kyriakou DS, Dambaki K, Niniraki M, Stathopoulos E. Ki-67 Proliferation Index. Am J Clin Oncol. 2004 Feb;27(1):8–13.
